# Recent Advances in Biosensor Technology for Early-Stage Detection of Hepatocellular Carcinoma-Specific Biomarkers: An Overview

**DOI:** 10.3390/diagnostics14141519

**Published:** 2024-07-15

**Authors:** Raja Chinnappan, Tariq Makhzoum, Momo Arai, Amro Hajja, Farah Abul Rub, Ibrahim Alodhaibi, Mohammed Alfuwais, Muhammad Affan Elahi, Eman Abdullah Alshehri, Lohit Ramachandran, Naresh Kumar Mani, Shugufta Abrahim, Mohammad Shabab Mir, Khaled Al-Kattan, Tanveer Ahmad Mir, Ahmed Yaqinuddin

**Affiliations:** 1 College of Medicine, Alfaisal University, Riyadh 11533, Saudi Arabia; tmakhzoum@alfaisal.edu (T.M.); marai@alfaisal.edu (M.A.); ahajja@alfaisal.edu (A.H.); farahaabulrub@gmail.com (F.A.R.); ialodhaibi@alfaisal.edu (I.A.); mohfuwaiz@gmail.com (M.A.); melahi@alfaisal.edu (M.A.E.); kkattan@alfaisal.edu (K.A.-K.); tmir@kfshrc.edu.sa (T.A.M.); 2 Tissue/Organ Bioengineering & BioMEMS Laboratory, Organ Transplant Centre of Excellence (TR&I-Dpt), King Faisal Specialist Hospital and Research Centre, Riyadh 11211, Saudi Arabia; aealshehri@kfshrc.edu.sa; 3 Microfluidics, Sensors & Diagnostics (μSenD) Laboratory, Centre for Microfluidics, Biomarkers, Photoceutics and Sensors (μBioPS), Department of Biotechnology, Manipal Institute of Technology, Manipal Academy of Higher Education, Manipal 576104, Karnataka, India; lohit.ramachandran@learner.manipal.edu (L.R.); naresh.mani@manipal.edu (N.K.M.); 4 Graduate School of Science and Engineering for Education, University of Toyama, 3190 Gofuku, Toyama 930-8555, Japan; shuguftawani23@gmail.com; 5 School of Pharmacy, Desh Bhagat University, Mandi Gobindgarh 147301, Punjab, India; shababmir123@gmail.com; 6 Lung Health Centre Department, Organ Transplant Centre of Excellence, King Faisal Specialist Hospital and Research Centre, Riyadh 11211, Saudi Arabia

**Keywords:** hepatocellular carcinoma, cancer biomarkers, biosensors, liver diseases diagnosis

## Abstract

Hepatocellular carcinoma is currently the most common malignancy of the liver. It typically occurs due to a series of oncogenic mutations that lead to aberrant cell replication. Most commonly, hepatocellular carcinoma (HCC) occurs as a result of pre-occurring liver diseases, such as hepatitis and cirrhosis. Given its aggressive nature and poor prognosis, the early screening and diagnosis of HCC are crucial. However, due to its plethora of underlying risk factors and pathophysiologies, patient presentation often varies in the early stages, with many patients presenting with few, if any, specific symptoms in the early stages. Conventionally, screening and diagnosis are performed through radiological examination, with diagnosis confirmed by biopsy. Imaging modalities tend to be limited by their requirement of large, expensive equipment; time-consuming operation; and a lack of accurate diagnosis, whereas a biopsy’s invasive nature makes it unappealing for repetitive use. Recently, biosensors have gained attention for their potential to detect numerous conditions rapidly, cheaply, accurately, and without complex equipment and training. Through their sensing platforms, they aim to detect various biomarkers, such as nucleic acids, proteins, and even whole cells extracted by a liquid biopsy. Numerous biosensors have been developed that may detect HCC in its early stages. We discuss the recent updates in biosensing technology, highlighting its competitive potential compared to conventional methodology and its prospects as a tool for screening and diagnosis.

## 1. Introduction

Liver cancer is the sixth most diagnosed cancer and the third leading cause of cancer-related death globally [1,2]. Hepatocellular carcinoma (HCC) is one of the most prevalent adult malignancies and the most common histological subtypes of primary liver malignancy. The patient population affected by HCC are typically those with pre-existing liver disease, most commonly cirrhosis [3]. Other causes for HCC include, but are not limited to, chronic hepatitis B or C infections, alcoholic liver disease, hereditary causes, and aflatoxin exposure [4]. The general pathophysiology of HCC involves mutations in the human genome at various sites, such as the promoter regions, TP53, and beta-1 catenin [3]. HCC has a significant impact on human health due to its poor prognosis, with a five-year survival lower than 20% in some instances [5]. Early diagnosis of HCC plays a significant role in improving the prognostic scores. Due to the numerous underlying pathophysiologies leading to HCC, patient presentation can vary, leading to difficulties in diagnosis. A majority of HCC patients are asymptomatic in the earlier stages of the disease, often leading to delayed diagnosis [6]. Some present with non-specific symptoms, such as right upper quadrant pain, weight loss, and, eventually, liver decompensation, all of which indicate a later stage of disease and a poorer prognosis. Despite many advanced detection methodologies, the burden of liver cancer remains significant [7,8].

Conventional methods for the diagnosis of HCC involve screening for various biomarkers implicated in the disease pathophysiology. Biomarkers are defined as “a characteristic that is objectively measured and evaluated as an indicator of normal biological processes, pathogenic processes or pharmacologic responses to a therapeutic intervention” [9]. In diagnosing HCC, they can provide a numerical value to underlying pathologies occurring in the liver, even without any other significant clinical presentations, providing physicians with an opportunity for early diagnosis, earlier management, and a more favorable prognosis. The first biomarker indicated for the screening of HCC is alpha-fetoprotein (AFP), a protein typically produced by the fetal yolk-sac and fetal liver [10]. 

While non-specific, elevated levels of AFP typically point towards an underlying liver pathology that may be malignant [11]. While AFP is typically elevated in patients with underlying HCC, a common issue is that upwards of 30% of patients, despite being infected with HCC, present with normal levels of AFP—a high specificity but low sensitivity. More specific isoforms of AFP for HCC diagnosis have been studied, with AFP-L3 being elevated in patients with smaller HCC tumors [10]. Various other isoforms have been discovered in the recent literature, but they are not used routinely in clinical practice, due to their demanding costs and high complexity. This gap in the clinical field of a particular and sensitive biomarker for early diagnosis has produced a high demand for further studies into biomarkers that are better for the surveillance of HCC. Various promising methods have been established for the sensitive detection of HCC biomarkers on the cancer cell surface or in the circulating body fluids [12,13,14]. Although these biomarkers are robust and efficient, they lack accuracy, sensitivity, and specificity in cancer diagnostic applications [7,15].

Moreover, these methods are costly, and some threads lead to exposure to radiation, which causes inconvenience to the patients. Though many biomarkers evolve constantly, no reliable biomarker is available for the specific and accurate diagnosis of HCC [16,17,18]. Liquid biopsy sampling is convenient, easy, and noninvasive or minimally invasive. Body fluids like urine and blood are great sources for detecting tumor biomarkers, including DNA, RNA, proteins, circulating tumor cells (CTCs), lipids, and microRNAs [7,19,20]. Though the detection of liquid-based biomarkers looks simple, there are many challenges. These include non-specific interaction, background signal suppression, or enhancement to achieve sensitive and accurate measurement. Some novel methods consisting of synergistic capture probes and molecular imprinting have been applied to improve the performance of the sensors. In addition, modern nanomaterials can be utilized to increase the sensitivity and specificity of the methods [19]. Alternatively, miniaturized biosensor devices can be used for the sensitive and quantitative detection of biomarkers [21,22]. 

**Figure 1:**
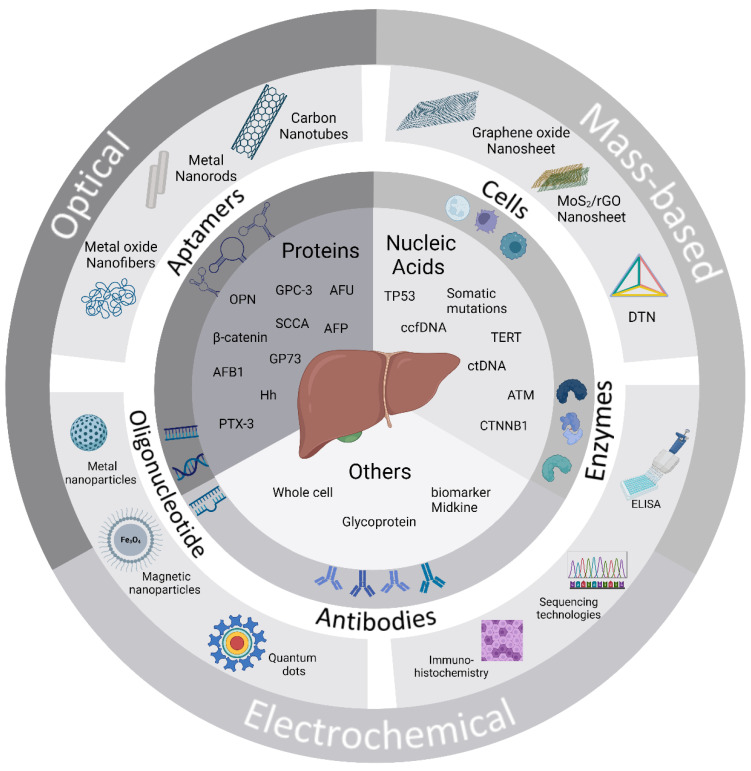
Pictorial representation of biosensing methodologies developed for the detection of HCC biomarkers. The diseased liver is shown in the center and the different types of target biomarkers such as nucleic acids and proteins are shown in the outer ring. Next to the biomarkers are the bioreceptors including antibodies, aptamers, enzymes, and nucleic acids used for biosensor development. The next compartment represents bioactive materials used as transducers (bio-recognition into signals) in different classes of biosensors such as optical, electrochemical, and mass-based.

In this review, we first summarize the notable advantages or disadvantages of conventional methods for diagnosing HCC (Table 1). Next, nucleic acid- and protein-based biomarkers for hepatocellular carcinoma are briefly introduced. The general concept of biosensing approaches for the ultrasensitive detection of hepatocellular carcinoma-specific biomarkers and the recent developments in biointerfacial strategies are also described (Figure 1). The linear ranges and detection limits achieved by different biosensing methods for hepatocellular carcinoma-specific biomarkers are also summarized (Table 2). Finally, the challenges and prospects of biosensors for hepatocellular carcinoma analysis are highlighted.

**Table 1:** Summary of advantages and disadvantages of the traditional methods for HCC diagnosis.

Diagnostic Methods	Advantages	Limitations	References
Positron Emission Tomography (PET)	Indicates both functional and anatomical information; To identify cancerous lesions; More accurate analysis of metastasis via lymph nodes.	Limited spatial resolution and cancerous and lesion detectability; Possible radiation exposure via intravenous administration.	[23,24,25]
Computed Tomography (CT)	Fast scan-reduced motion artifacts; Accurate spatial information.	Suboptimal soft tissue imaging; Risk of radiation exposure; Limited functional information.	[24,26]
Magnetic Resonance Imaging (MRI)	Soft tissue imaging reveals more information; Reduces the ionizing radiation exposure; The gadolinium contrast agent used in MRI causes fewer allergic reactions than iodine contract agents in X-rays and CTs.	Internal and external metal objects due to interference with the magnetic fields; High cost; Time-consuming;.	[24,27]
Magnetic Resonance Spectroscopy (MRS)	Detailed soft tissue imaging; Limited radiation exposure; One can obtain biological, anatomical, physiological, and metabolic information.	Time-consuming; Highly expensive; No detailed anatomical information.	[28,29]

## 2. Biomarker and Biosensors

**Figure 2:**
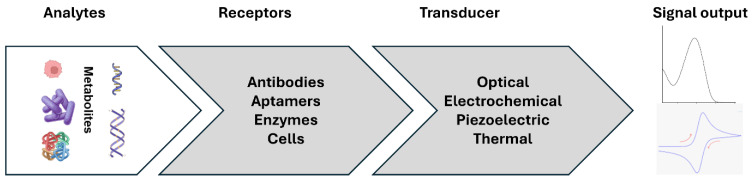
Illustration of working principles of biosensors. The red arrows represent the oxidation and reduction directions.

Clinical biomarkers are any biomolecules that are measurable by quantity, structure variation, or chemical or biochemical reaction and can detect or predict the outcome of the diseases. Various biomolecules such as DNA, RNA, enzymes, proteins, hormones, or metabolites are used as biomarkers for the early diagnosis of HCC. These biomarkers are classified into nucleic acid-based, protein-based, and other biomarkers such as metabolites and whole cells. The significant role of biomarkers in the HCC and biosensors developed for early-stage detection is discussed in each sub-section. A biosensor is a device consisting of three major components: a target recognition element, transducer, and signal output/amplifier. In the first step, the recognition element recognizes the specific target from the sample, triggering a unique biological response that can be converted into a signal by the transducer. Finally, this signal is amplified into the measurable output signal. The biosensors are classified into three major types, optical, electrochemical, and mass-based, based on the transducer used in the sensor design, as shown in Figure 2. Several reports have been developed and summarized for diagnosing HCC biomarkers [12,30,31,32,33,34].

## 3. Nucleic Acid Biomarkers

Hepatocellular carcinoma (HCC) is one of the most common malignancies worldwide, with a high incidence, frequent recurrence, rapid progression, and poor prognoses [35,36]. Hepatocarcinogenesis relies heavily on genetic and epigenetic aberrations triggered by etiological factors and tumor progression [37]. Due to its severity and prevalence, there is a rising yet unmet need for biomarker detection in the early-stage diagnosis of HCC [38]. Although biopsies remain an essential diagnostic tool in the early detection of HCC, their repeated application is contraindicated due to potential complications [39]. “Liquid biopsies” allow for the analysis of non-solid biological tissues, providing a far less invasive alternative to assessing the real-time evolution of cancers [40]. Despite the lack of satisfactory diagnostic tools through blood-based media, circulating tumor DNA (ctDNA) carrying cancer-specific genetic alterations provides a gateway for noninvasive liquid biopsy usage in the early detection of HCC [41]. ctDNA assays have seen considerable improvement, transitioning from the simple Sanger method to the more highly sophisticated and sensitive employment of emulsion, digital droplet polymerase chain reaction (ddPCR), amplification and magnetics technology, and beads [4]. In a total of 171 detected genes, the most frequently noted endogenous alterations were TP53 (54%), CTNNB1 (42%), TERT (42%), and ATM (25%) [39]. Other, less frequent genes include tyrosine-metabolism-related genes [36], AXIN1, RPS6KA3, ARID2, MLL2, TSC1/TSC2, ARIDA1A, and KEAP1 [42].

### 3.1. Tumor Protein (TP53) Gene

TP53 is, physiologically, a tumor suppressor gene. However, this gene develops strong oncogenic potential by acquiring mutations, most commonly of the missense moiety [43,44]. TP53 is the most common mutation in HCC due to its relation to the immune microenvironment [45]. The CRISPR knockout of TP53 in hepatic tissue has maintained frequent neoplastic changes and is thus a frequent area of research [46]. Despite its prevalence, conventional sequencing and immunohistochemical analyses are often contradictory, urging the emergence of ultrasensitive biosensing technologies, including TI-SPR-1 [47], an electronic DNA hairpin molecular beacon [43], and aligned multi-walled carbon nanotubes (A-MWCNTs)/Au nanoparticles [48]. These biosensors, amongst many others, provide a more straightforward and cheaper method of DNA mutation detection over traditional hybridization assays, typically with greater sensitivity, selectivity, reproducibility, and reduced assay analysis time [49]. The TP53 gene inactivation is common in most of the cancer types; therefore, most of the research is related to the mutation of these genes associated with the cancer progression and therapy response. The specific mutations in the L2 and L3 zinc-binding domains are significantly linked to therapy and the drug resistance in most of the cancers. The DNA biosensors use SPR and Texas instruments’ spreeta chips from the cDNA of the target domains. The sensor was developed based on the binding behaviors of mismatch repair protein MutS and single-strand binding proteins. To enhance the sensitivity to mutations three-fold, mismatch binding protein MutS and single-strand binding protein (SSB) were applied after probe hybridization. MutS did not bind to mismatch oligonucleotides. However, SSB can bind to unhybridized ssDNA probes. The difference in binding resulted in a three-fold increase in the sensitivity of the sensors [47]. Sun et al. were able to display not only high sensitivity but also high specificity for TP53 mutation hotspots through the usage of sulfhydryl-ended hairpin DNA probes tagged with methylene blue, achieving an LOD of 10 nmol L^−1^. By attaching these DNA probes onto a gold electrode, hybridization reactions result in significant signals detectable through voltammetry, showing enhanced capacities for recognizing base signal mismatches and single nucleotide polymorphisms (SNP) of the TP53 gene [43]. An even lower LOD of 1.0 × 10^−17^ M was achieved through A-MWCNT synergistically attached to Au nanoparticles without amplification, significantly increasing sensor sensitivity, stability (14 days), and reproducibility (RSD = 2.1%). These A-MWCNT/AuNPs serve as working electrodes for the label-free, sensitive detection of the TP53 gene mutation [48]. Further studies need to be performed on the efficacy and efficiency of these modalities to assess their exact specificities and sensitivities better.

### 3.2. Catenin Beta-1 (CTNNB1)

Catenin beta-1 (CTNNB1) codes for β-catenin, a major subunit of a signaling molecule in the WNT pathway, which controls many biological processes [50,51]. CTNNB1 mutations are typically at or near phosphorylation sites, inhibiting β-catenin phosphorylation and, thus, chronic activation [51]. It has been demonstrated that HCC patients presenting with Wnt/β-catenin pathway mutations, including CTNNB1, are less likely to respond to immunotherapy, highlighting the urgency of their early detection [52].

Biosensing modalities remain sparse. However, ddPCR may provide an invaluable amplification of otherwise infrequently mutated alleles, making them more easily recognizable amongst the vast array of somatic alleles in tumor samples and plasma [52]. ddPCR might be combined with the Sanger method to detect frequencies of DNA aberrations, which is significant when comparing cirrhotic versus non-cirrhotic HCC patients through much more highly sensitive and specific biosensing modalities. As earlier studies showed hotspot mutations in exam 3 of the CTNNB1 gene, standardized primers can amplify ultra-high sensitivities and precisions [53].

Alternatively, a newly established bimolecular fluorescence complementation (BiFC) biosensor system may be used to better visualize the peak in activity of CTNNB1 during the S and G2 phases of the cell cycle, providing unique insights into sites of inhibition opportune for future projects. To yield a signal, the crystal structure of β-catenin was coupled with TCF3 to produce a functional biomolecular fluorescent complex. In order to test the specificity of this interaction and the BiFC biosensor, two mutated moieties of TCF3, TCF3ΔNLS and TCF3ΔN, were recruited for their competitive binding, deducing that the BiFC reduction signal was indeed specific [54].

### 3.3. Telomerase Reverse Transcriptase (TERT)

TERT (Telomerase Reverse Transcriptase) mutations have been described as “gatekeepers” in early HCC transformation, leading to a consecutive acquisition of hypermethylation in cirrhotic tissue [55]. Hepatitis B virus (HBV) infection has a predilection to integrate into the TERT promotor region, a hotspot for dysregulation of the standard hepatocytic transcription, contributing to developing the neoplastic process [42]. The detection of TERT in early HCC might point towards a worse prognosis and eventual progression into a full-blown malignant transformation in advanced HCC [42]. Thus, the practical usage of assisting biosensing technologies is essential in early detection, traditionally carried out through whole-exome sequencing [42].

HBV integration site detection has seen a substantial progression from previously used Southern blot platforms into much more rapid inverse polymerase chain reactions; however, there remained a weakness in detecting said HBV integration sites at the nucleotide level [42]. Through the development of multiple next-generation sequencing (NGS) genomic platforms, including RNA sequencing (RNA-seq), whole-genome sequencing (WGS), and capture sequencing (Cap-seq), these detections are made much more feasible and specific [56]. Although there remains a lack of suitable methods for the detection of ctDNA in this context, circulating cell-free deoxyribonucleic acid (cfDNA), of which ctDNA is a fragment, may provide a unique opportunity to analyze hotspots of point mutations of the TERT promotor through means of wild-type blocking polymerase chain reaction (WTB-PCR) coupled with Sanger sequencing [38,57].

Although ELISA and PCR detection methods are less invasive and highly sensitive, their cost and prolonged detection times make them less favorable for frequent usage [43]. Akuta et al. resorted to an analysis of substitutions of cytosine for thymidine at position 228 (C228T), a hotspot mutation of TERT, using a wild-type blocking polymerase chain reaction (WTB-PCR) combined with Sanger sequencing to determine the lowest LOD of WTB-PCR [27]. On the other hand, cap-seq provides a more advantageous reading of the HBV-containing junctional DNA fragments via curated capture probes. However, these probes are not designed to detect smaller HCCs, hindering reading potential and viability. With the improvement in cap-seq’s generations, it might prove to be a valuable tool for the detection of even minute traces of HBV-associated TERT in blood, as well as low levels of vh-chimera DNA from clonal hyperplasia with indeterminate potential (CHIP) [56].

### 3.4. Ataxia Telangiectasia Mutated (ATM)

Ataxia telangiectasia mutated (ATM) protein kinase plays a significant responsive role in signaling a damage response towards DNA double-strand breaks, which promotes mutated cell longevity [58]. Much of the autophosphorylation effects of ATM are exemplified in a protein cascade and can be detected through immunoblotting, which displays a separation of ATM from other antibodies utilizing primary and secondary antibodies, immunofluorescence, mass spectrometry, and ChIP—which employs IgG beads directed against the ATM protein or kinase assays [58]. A close inspection of ATM expression through immunohistochemistry yielded notably elevated levels of HCC compared to nearby normal tissue [59].

Kinase assays in vitro are routinely used to assess ATM protein activity alongside immunoprecipitation–Western blotting and a fluorescence resonance energy transfer (FRET)-based biosensor [60]. Most kinase assays are, however, limited in their ability to detect protein activity in intact cells or in situations where multiple signals are simultaneously emitted from the same cell; mass spectrometry and peptide-based biosensor substrates provide an optimal detection for the intracellular activation pathway following the autophosphorylation cascade [61,62]. It has subsequently been found that immunoblotting analyses of nuclear, rather than whole-cell, lyases provided far more sensitive and specific results, of which further studies must be conducted to determine the exact values [63].

### 3.5. Methylated DNA

DNA methylation, in which methyl groups are added to carbon-5 cytosine–guanine dinucleotide (CpGs) residues via methyltransferases without changing the DNA sequence, plays an essential role in the cascade of epigenetic modifications regressing towards HCC, with superior sensitivity and specificity as biomarkers for the early detection of HCC compared to those conventionally used for surveillance [64,65]. A developed methylation-sensitive high-resolution analysis (MS-HRM), coupled with Lactate Dehydrogenase B (LDHB) analysis, yielded a 57% sensitivity for HCC detection, whereas alpha-fetoprotein (AFP) had a 45% sensitivity at a similar specificity [66]. Where the sequencing of thousands of allele sequences might be necessary to determine mutational genetic patterns specific to HCC, the screening of as few as five aberrant methylation biomarkers of samples taken from HCC patients displayed a 95% specificity and were displayed in 96% of DNA samples taken from neoplastic tissue [42]. Furthermore, methylation profiles of cfDNA-based HCC screening models of liquid biopsies demonstrated a sensitivity of 86% and specificity of 98% in a training cohort and a sensitivity of 84% and specificity of 96% in an independent validation cohort [66].

Since the differentiation between non-methylated cytosine and methylated cytosine is subtle, it may be difficult to detect through quantitative real-time polymerase chain reaction (qRT-PCR) alone. This led to the development of more intricate detection modalities catered towards that discrimination, an example of which is bisulfide sequencing [64]. In an acidic, warm environment, bisulfide conversion first involves transforming cytosines into sulfated cytosines, which are then converted to sulfonated uracils catalyzed by a deamination reaction, finally yielding uracils via alkaline desulfonation [67]. Through bisulfide sequencing, methylated DNA residues remain intact after DNA extraction and treatment, opening the gateway for amplifying these remnants via PCR, such that Sanger or Illumina short-read sequencing methods can easily detect them. Although bisulfite sequencing remains the only method capable of localizing DNA methylations, it has its own disadvantages, exemplified by DNA breakages, degradation, and a lack of specificity [68,69].

As such, establishing a more suitable biosensing modality was crucial, leading to the construction of a two-part biosensor made up of a gold electrode and nanocomposite (AuNPs/rGO/g-C3N4). In order to exercise its DNA methylation sensing capabilities, target DNA was captured by a probe DNA and tagged by gold nanoparticles (AuNPs). These tagged target DNA molecules were subsequently recognized by non-methylated cytosine residues, producing an observable alteration in electrochemical signaling. For short-sized DNA methylations, an LOD of 0.74 fM was observed. For longer DNA methylations, an LOD of copy number 10^3^ was achieved. This approach was also stated to have a high sensitivity and specificity [68]. Lin et al. developed a sensitive ECL method for detecting DNA adenine methylation methyltransferase (Dam MTase). Ru(bpy)_3_^2+^ incorporated a metal–organic framework as an ECL indicator. Dam MTase restriction endonuclease (DPnI) cleaves hairpin DNA (HP) and releases the Dam. The ssDNA of the loop binds to the crRNA and activates the CRISPR/Cas12a trans cleavage activity, which leads to the non-specific cleavage of ssDNA-Fc modified on Ru-MOF. The shredding of ssDNA-labeled Fc separates the Fc from the signal unit and restores the ECL signal. This sensing method can achieve a lower LOD of 23.4 mU/mL for DNA adenine methylation methyltransferase (Dam MTase) in serum samples [70].

**Figure 3:**
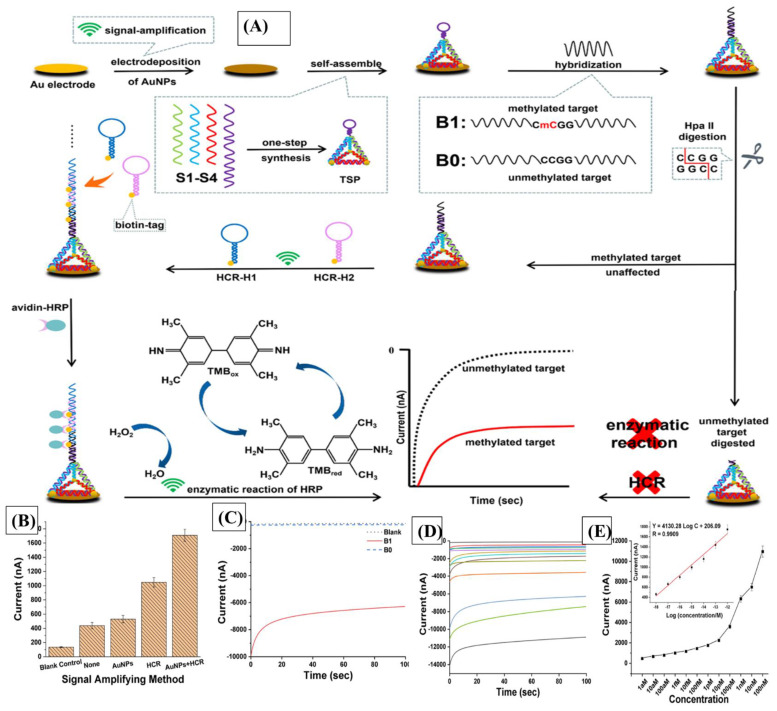
Schematic depiction of the biosensing and multiple signal amplification strategies for detecting DNA methylation (**A**). The graph shows the validation test response corresponding to the signal amplification function (**B**). Electrochemical signal responses (chronoamperometric measurements) of the blank control (dot), unmethylated target (dash), and methylated target (solid) (**C**). Chronoamperometric response signals of methylated targets in a concentration-dependent manner (from top to bottom) (0 aM, 1 aM, 10 aM, 100 aM, 1 fM, 10 fM, 100 fM, 1 pM, 10 pM, 100 pM, 1 nM, 10 nM, and 100 nM) (**D**)—the dose−response curve of different target concentrations. The inset shows the linear relationship between the chronoamperometric signals and the logarithm of target concentrations from 1 aM to 1 pM (**E**). (N = 3). Adapted from ref. [71] with copyright permission.

Electrochemical biosensors can broaden the detection horizon further, with an LOD of about 0.93 aM, showing ideal reproducibility conditions and specificity. Chen et al. developed an ultrasensitive electrochemical biosensor capable of DNA methylation detection utilizing an AuNP-coated disk anchoring a loop of DNA probes. An enzyme, HpaII, was used to restrict the digestion of unmethylated targets. Once met with a methylated hybridization palindrome sequence, digestion would terminate, and the methylated DNA would not be washed off. Finally, HCR and HRP amplifications would be performed to yield high enough signals for detection [71] (Figure 3. Mitigating the otherwise cumbersome steps of amplification, enzymatic digestion, or even bisulfite treatment, an alternative electrochemical biosensor was constructed based on toehold-mediated strand displacement reactions and yielded an LOD of 0.075 pM, where one detection cycle can be completed within 1 h (60 min). The biosensor was developed by utilizing peptide nucleic acid (PNA) probes, which invade methylated DNA far more readily than its unmethylated counterparts, producing significant electrical signals upon binding [69]. Despite the presence of multiple biosensing modalities, further studies must be conducted to pinpoint exact DNA methylation residues and produce more sensitive and specific tests for rapid detection in early HCC.

### 3.6. MicroRNA/Long Noncoding RNA

**Figure 4:**
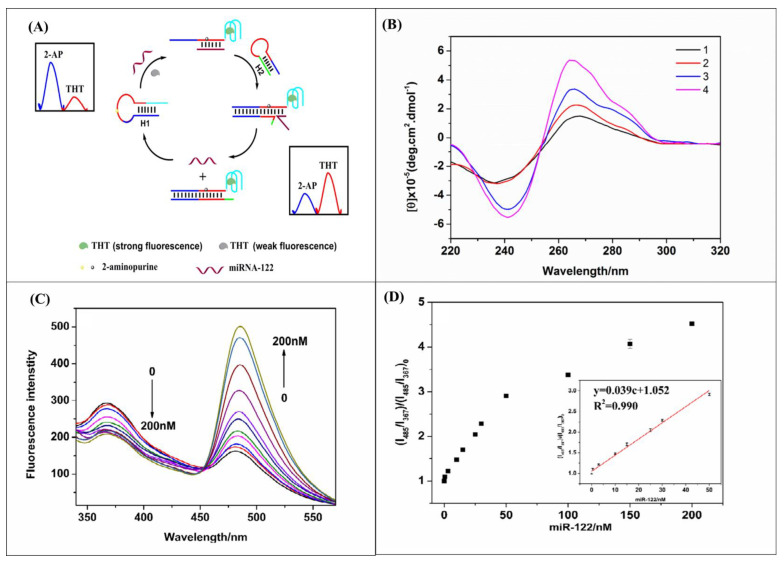
Schematic representation of the ratiometric probe for the detection of miRNA-122 (**A**). CD spectra of curves (1) H1, (2) H1 + miRNA122, (3) H1 + H2, and (4) H1 + H2 + miRNA-122 (**B**). The fluorescence signal response of the probe in the presence of miRNA-122 is concentration-dependent (0, 0.5, 3, 10, 15, 25, 30, 50, 100, 150, and 200 nM) (**C**). The linear plot of the PL intensity ratio (I485/I367)/(I485/I367) versus the concentration of miRNA-122 (**D**). (N = 3). Adapted from ref. [81] with copyright permission.

MicroRNAs (miRNAs) are short strands of noncoding RNA molecules that target mRNA’s 3′ end UTR to regulate gene expression. They mainly inhibit the translation of target genes by degradation through base pairing, one of the essential regulators of post-translational modification [72]. A lot of cancers and diseases are related to the regulation of these RNA molecules [73]. Studies have shown that its detection can be used as a biomarker for hepatocellular carcinoma [74]. It was found that they played a significant role in regulating important cellular processes such as apoptosis, angiogenesis, migration, and proliferation [75]. Thus, it can be used as an essential biomarker to diagnose liver cancer from other diseases like hepatitis or cirrhosis. They have a high stability and extractability from saliva and urine; some of them can even be screened to help diagnose HCC in its earlier stages, making them a reliable marker for diagnosis [76]. Sayed et al. used circulating miRNA as a biomarker for the early detection of HCC in Egyptian patients by machine learning algorithms [77]. Several detection methods are being studied, including electrochemical signals, fluorescence, resonance light scattering, and nanoparticles [78,79]. A highly selective and sensitive AgNP and propolis-modified carbon paste electrode (APCPE) was used to detect miRNA by measuring electrocatalytic activity. The target miRNA let7a was detected using complementary miRNA ssDNA as a capturing probe. The EIS signal from the probe-DNA-modified electrode was used to detect the target miRNA. It was shown to detect miRNA at an LOD of 10^−3^ femtomoles within 30 min [80]. Hairpin-assembly-based turn-on ratiometric fluorescence was used to detect miR-122 using 2-aminopurine (2-AP) and thioflavin (ThT) as fluorescence probes. In this design, the hairpin DNA (H1) was linked with a complementary strand of miR-122 and a G-quadruplex forming sequence. In the presence of the target, a part of the hairpin DNA for duplex and G-quadruplex leads to the diminished fluorescence of 2-AP. The ThT forms a complex with g-quadruplex and increases the fluorescence of ThT. In the next step, another hairpin DNA (H2) was introduced to release the target miRNA, which further hybridized and the cyclic processes continued for the signal amplification. This method was tested in the linear range of 0.5 to 50 nM, and the LOD was 72 pM [81] (Figure 4).

Specific miRNAs in the serum, such as miRNA-130b and miRNA-15b, increased in hepatocellular carcinoma. The sensitivity and specificity of miRNA-130b were 87.7% and 81.4%, respectively, whereas it had a high sensitivity of 98.3% and a low specificity of 15.3% for miRNA-15b. Thus, it was found that a combination of these markers may help diagnose early stages of liver cancer rather than separately [82].

Other important miRNA biomarkers include miRNA-122, miRNA-21, and miRNA-224, which are all elevated in hepatocellular carcinoma compared to normal liver cells. miRNA-122, to be specific, was shown to have a significantly higher level in early-stage HCC [83]. Graphene-based electrochemical biosensors for detecting HCC-specific biomarkers, including miRNAs, exosomes, and other nucleic acid and protein biomarkers, have been discussed extensively [84]. The simultaneous label-free detection of miR-122 and AFU by the resonance light scattering (RLS) method was developed using methyl violet as a fluorescence probe, where the RLS intensities were compared with normal cells from carcinoma cells. The LOD of miRNA was 98 pM [85]. The combination of DNA-quadruplex-forming DNA sequences and the DNA sequence of miR-122 formed a duplex with the cDNA miR-122. In the presence of the target, the complementary sequence hybridizes with miR-122 and the ssDNA to form a hybridized chain of G-quadruplex (G-nanowire) by adding K^+^ and Mg^2+^ ions. It forms self-assembly to form long filamentous G-wires. The LOD of this method was 6.1 pM at an assay time of 2 h [86]. Moreover, using electroactive Prussian Blue (PB) on a graphene oxide (GO) electrode and measuring the electroactive PB/GO current were effective, with an LOD of 1.5 fM in 60 min. This was one of the methods of detection showing excellent selectivity, reproducibility, and good stability [87].

Wu et al. designed a magnetically assisted sandwich-type surface-enhanced Raman scattering (SERS)-based biosensor, a highly sensitive and specific method. This multiplex detecting system can simultaneously detect three different HCC-related miRs (miR-21, miR-122, and miR-223). The LOD of miR-21, miR-122, and miR-223 are 311aM, 349 aM, and 374 aM, respectively [88]. The level of miR-21 decreases after hepatectomy; however, the levels of miR-122 and miR-223 increase after hepatectomy. A second miRNA associated with early-stage HCC is miRNA-16. Using a dual-aptamer hairpin DNA oligonucleotide to bind to miRNA-16, its electrochemical signal can be detected, allowing for the measurement of its levels. Its LOD was 0.14 nM [89]. Thus, a combination of miRNA-16 and miRNA-122 could be used to detect HCC in its early stages.

miRNA-21 is another important biomarker for the detection of HCC. There are different detection methods, each exhibiting a high sensitivity and specificity. It can be measured using a multifunctional magnetic nanoparticle probe for electrochemical detection (LOD: 0.46 fM), a target-recycled enzyme-free CHA with the detection of quantum dots (LOD: 200 amol), and a hybridization chain reaction lateral flow assay to detect the amount of miRNA-21 (LOD: 0.3 fM) [90,91,92]. Newer methods of detection have also been on the rise.

Recently, zip nucleic acids were also used to detect miR-34a, a known tumor suppressor gene in HCC. It was detected using a streptavidin-coated magnetic bead (MB) surface, and then the guanine oxidation signal was measured. HCC would have a lower signal. The LOD was found to be 0.87 µg/mL [93]. Furthermore, U-shaped biosensors utilizing wavelength shift and transmission loss can also be used to detect miRNA-133a with excellent sensitivity and specificity, 27.352 dB/log ng/mL and 0.886, respectively, and an LOD of 0.0133 ng/mL as these levels were found to be lower in tumor cells compared to control cells [94]. Wang et al. developed an electrochemical genosensor utilizing CuNPs to detect miRNA-222. The sensor relies on DNA-templated copper nanoparticles (CuNPs) as a signaling probe. MiRNA-222 was chosen as the model analyte [95]. The probe was derived from two distinct oligonucleotides with complementary bases through a hybridization chain reaction, forming long DNA concatemers as templates. The reaction of ascorbate with copper sulfate generated the Cu NPs. The genosensor demonstrated a low LOD of 0.03 fM and a linear detection range of 0.5 fM to 70 nM. Li et al. developed a sensor for detecting HULC using Au@Ag core–shell nanoparticles/graphene quantum dots (Au@Ag/GQDs) [96]. The electrochemiluminescent (ECL) sensor demonstrated high sensitivity. Using Au@Ag core–shell nanoparticles in conjunction with graphene quantum dots (GQDs) as a signal indicator allowed for enhanced performance. The sensor reached an LOD of 0.3 fM with a linear detection range from 1 fM to 5 nM.

Zhang et al. developed a sensor for detecting miRNA-141 using Fe_3_O_4_ NPs, featuring one of the lowest LODs [97]. The system comprises an indium tin oxide cathode and a graphene oxide/gold nanoparticle/glucose oxidase anode. DNA encapsulates a redox probe of [Fe(CN)_6_]^3−^ inside porous Fe_3_O_4_ nanoparticles. When the target miRNA is present, a hybridization occurs between miRNA and DNA, releasing [Fe(CN)_6_]^3−^. The duplex-specific nuclease is employed to initiate target recycling amplification, releasing more redox probes and a significant increase in open-circuit voltage. The sensor achieved an LOD of 1.4 aM and demonstrated a linear detection range from 10 aM to 10 fM.

Miao et al. developed a sensor for the detection of miRNA-141 using CuNPs. This study introduces an amplified electrochemical method for miRNA detection employing T7 exonuclease (exo) and copper nanoparticles (CuNPs) [98]. Double-stranded DNA modified on the electrode surface serves as the template for the in situ synthesis of CuNPs, which act as excellent electrochemical signal sources. Two cycles of DNA cleavage reactions are meticulously designed based on the catalytic activity of T7 exo, occurring both in the solution and at the electrode surface. These two cycles are integrated for cascade signal amplification. Target miRNA initiates the first cycle, and its product triggers the second cycle, destroying the template on the electrode for CuNP synthesis. Consequently, the electrochemical signal decreases and reflects the level of the initial miRNA. The sensor achieves an LOD of 4.5 × 10^−17^ M and demonstrates a linear detection range from 10^−16^ to 10^−13^ M [98].

Xu et al. developed an electrochemical genosensor for detecting miRNA-21 and miRNA-155 using a tetrahedron DNA nanostructure (TDN) [99]. In this design, the DNA circle capture probe is anchored at the top of the TDN, allowing for the simultaneous recognition of miRNA-21 and miRNA-155 through multiple target recognition domains with the assistance of Helper strands. This configuration triggers a mimetic proximity ligation assay (mPLA) for capturing the beacons ferrocene (Fc)-A1 and methylene blue (MB)-A2, facilitating the detection of multiple miRNAs. The sensor achieves an impressively low LOD of 18.9 aM for miRNA-21 and 39.6 aM for miRNA-155, demonstrating high sensitivity. It exhibits a wide linear detection range spanning from 0.1 fM to 10 nM. Guo et al. developed a sensor for detecting miRNA-122 using hybridization chain reaction (HCR) and hairpin DNA (hpDNA). This sensitive electrochemical assay was designed to detect exosomal miRNA-122 (miRNA-122) [100]. The gold electrode surface was initially modified with immobilized hpDNA probes. Upon the presence of miRNA-122, the hairpin structure of hpDNA unfolded, initiating the HCR through the cross-opening and hybridization of two helper DNA hairpins. The resulting long-nicked double helixes from HCR effectively captured more RuHex, enhancing the signal for differential pulse voltammetry (DPV). This innovative sensor achieves an impressively low LOD in the attomolar range and covers a wide linear detection range spanning nine orders of magnitude.

Xu et al. developed an ultrasensitive and specific multiple-miRNA detection strategy capable of detecting miRNA-122 and miRNA-21 [101]. This innovative approach relies on the strand displacement amplification (SDA) reaction and analogical catalytic hairpin assembly (ACHA) reaction. The target miRNA selectively binds with the template, initiating the SDA reaction to produce abundant amplification products (triggers). These triggers then participate in the ACHA reaction on the electrode surface, resulting in a decreased electrochemical response. The sensor exhibits an impressively low LOD of 0.012 fM for miRNA-122 and 0.075 fM for miRNA-21, with a linear detection range spanning from 0.1 fM to 10 fM.

Meng et al. developed an electrochemical impedimetric biosensor for detecting miRNA-21, utilizing a hybridization chain reaction (HCR) amplification strategy [102]. This biosensor enables the quick, sensitive, and specific detection of miRNA-21 by monitoring changes in the interfacial properties of the electrode in real-time. The HCR amplification is initiated by two sequences, H1 and H2, leading to the formation of many linear DNA concatemers. The resulting changes in the interfacial properties, precisely the interfacial charge transfer resistance difference (Rct), are probed through electrochemical impedance spectroscopy (EIS). The device achieves a remarkable LOD of 4.63 fM.

In the same year, Luo et al. developed a highly sensitive device for detecting miRNA-21, surpassing the LOD achieved by Meng et al. [102]. This ratiometric electrochemical DNA biosensor is based on a locked nucleic acid (LNA)-modified “Y” shape-like structure [103]. The sensor operates on the principle that in the presence of miRNA-21, the LNA-assisted strand displacement reaction is triggered on the “Y”-like structure. This activation induces a structural change, enhancing the signal ratio by reflecting the distances between the electrode surface and two electroactive molecules labeled on the “Y”-shape-like structure. The device achieves an impressive LOD of 2.3 fM.

Yu et al. developed an innovative device for detecting miRNA-21 utilizing DNA nanospheres. This ultrasensitive electrochemical biosensor integrates a nicking endonuclease-assisted primer exchange reaction (PER) cascade amplification with a DNA nanosphere (DNS)-mediated electrochemical-signal-enhanced system [104]. The cascade amplification begins with a nicking endonuclease that selectively cleaves specific DNA substrates, leading to a highly amplified production of single-stranded DNA fragments (ssDNA) from the target. Subsequently, the PER cascade, powered by strand-displacing polymerase, generates a significant amount of nascent single-stranded connector DNA (cDNA) through the ssDNA triggering of the dumbbell probe (DP), achieving the cascade amplification of the target. The device achieves an impressive LOD of 0.58 aM and has a linear detection range from 1 aM to 0.1 nM.

Park et al. developed a mass-based piezo-sensor for detecting miRNA-21, utilizing AuNp quartz crystal microbalance (QCM) biosensors [105]. These sensors were designed to achieve the sensitive and specific detection of miR-21 by forming miRNA-21-DNA hybrid duplexes and allowing the non-specific intercalation of surface-modified pyrene molecules. The biosensors demonstrated high selectivity for miR-21 over other miRNAs, attributed to the specific hybridization between miRNA-21 and gold nanoparticle (AuNP)-conjugated complementary oligonucleotides of miRNA-21. The method achieved high sensitivity by forming intercalated complexes on the surface, coupled with subsequent gold staining signal amplification. The LOD was determined to be 3.6 pM, and the linear detection range spanned from 2.5 pM to 2.5 μM [105].

Premaratne et al. developed a device for miRNA-21 detection that utilized gold nanoparticles (AuNps) [106]. Their approach involved utilizing a surface-immobilized capture oligonucleotide probe, which selectively formed hybrids with the target oligonucleotide, specifically the miRNA-21 mimic. Gold nanoparticles (50 nm) were linked with the target oligonucleotide to boost detection sensitivity, employing quartz crystal microbalance (QCM) and Surface Plasmon Resonance Imaging (SPRi) microarray techniques. The device exhibited a detection limit (LOD) of 28 fM, showcasing a notably lower sensitivity threshold than the outcomes reported by Park et al. [105]. Several biosensing strategies such as fluorescence spectroscopy, SERS, ECL, and different kinds of electrochemical methods have been applied for the detection of miRNs. In the LOD, all of the methods were in the range of sub-nM to aM. Among these methods, electrochemical techniques showed outstanding performance in terms of LOD (in aM).

### 3.7. Circulating Tumor Nucleic Acid Biomarkers

Sequencing technologies have also improved the detection of HCC by using serum nucleic acid markers [107]. Detecting DNA modifications in the serum is very useful in detecting early HCC stages because any alteration affecting the cell cycle, apoptosis, or gene maintenance could regulate the progression of harboring tumor cells. The detection of circulating cell-free DNA (ccfDNA) and its tumor-derived fraction (ctDNA) is a promising method using liquid biopsy as studies showed that the level of ccfDNA correlates with the stage of HCC. Somatic mutations such as p53, wnt B-catenin, the RASSF1A gene, the mGSTP1 gene, and TP53 249T can also be detected in ccfDNA, which saves the patient from an invasive liver biopsy. This noninvasive method could be utilized instead to allow an easier method of detection as well as a more convenient way of analyzing HCC, as it is a heterogeneous disease, where only some parts of the liver are affected [107].

Thus, liquid biopsy is a newly developed noninvasive method of detecting ctDNA, a dynamic real-time nucleic acid biomarker of HCC. In addition, sequential analysis for gene mutations can be performed on the ctDNA to identify the tumor progression further. Ultra-deep targeted sequencing showing cis-mutations confirms whether the detected DNA is from a tumor origin, so detecting somatic mutations in ctDNA essentially aids in diagnosing HCC [108]. For example, 401 methylation markers were detectable similarly in the tumor cells of patients with HCC, matching the ccfDNA sequences from plasma. This set of markers was then analyzed in the ccfDNA of 715 patients with HCC and compared with 560 healthy individuals, which then confirmed and allowed the emergence of a diagnostic score including ten methylation markers within the ccfDNA for HCC [109].

Another advantage is that the ctDNA harbors the exact contribution of different tumor clones and metastatic states, which signifies the heterogenetic nature of the tumor. One way to analyze this is by PCR-based techniques, which were found to have a higher sensitivity and specificity than the traditional Sanger sequencing [42]. Currently, digital next-generation sequencing is taking over widely since it also allows their detection in a very diluted DNA sample [42]. This improves the early diagnosis of HCC and greatly improves its management.

## 4. Protein Biomarkers

### 4.1. Alpha-L-Fucosidase (AFU)

Alpha-L-Fucosidases (AFUs) are mammalian lysosomal glycosidases involved in the degradation of glycoconjugates in many body tissues and cells [110]. The diagnostic importance of AFU in HCC has increased in recent years. AFU is a potential additional marker in the diagnosis of HCC, with a high sensitivity and specificity of 90 and 97.5%, respectively, at a cut-off of 2.3005 μmol/L/min [111,112]

**Figure 5:**
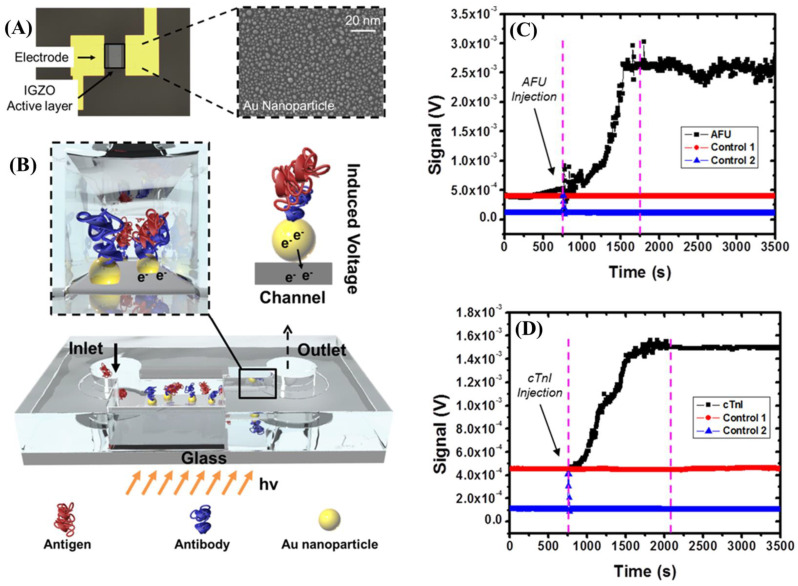
(**A**) Development of chip-based plasmonic biosensor modified with AuNPs on the surface of the special nanostructures with a wide-bandgap semiconducting material, InGaZnO (IGZO), and active layer between two gold electrodes. The inset shows the SEM image of AuNPs. (**B**) Illustration of cross-section of the parallel flow through biosensor with a microfluidic channel. Inset displays the induced electron flow from the AuNP through antigen–antibody interaction. Real-time measurement of (**C**) AFU and (**D**) cTnI detection with blind protein control (red line) and gold nanoparticle control (blue line). In addition, 4 U/L AFU and 0.1 ng/mL cTnI were prepared in PBS buffer solutions and injected into the biosensor at 750 s, respectively. The signal was stabilized at around 1750 s. Adapted from ref. [113] with copyright permission.

The use of biosensors to detect AFU has increasingly significant implications. Ultrasensitive plasmonic biosensors could detect AFU from whole human blood. One biosensor converted plasmonic absorption to electrical currents, allowing AFU detection in real-time. The detection limit was 0.016 U/L with high sensitivity [113] (Figure 5). Another ellipsometry biosensor showed a remarkable potential for the joint detection of HCC biomarkers. Using imaging ellipsometry, the biosensor could detect AFP, AFU, and ferritin within 60 min with an LOD of 1 U/L for AFU.

Moreover, the joint detection of AFP and AFU using the biosensor had a higher AUC (area under the curve) compared to clinical methods of detection, which provided a higher detection specificity for HCC [114]. Similarly, ion identification using a potentiometric biosensor for detecting AFU has shown remarkable findings. The biosensor used a PVC membrane sensor, which measured the concentration of 2-chloro-4-NP using a 2-chloro-4-NP-rhodamine B ion pair. This method allowed the detection of AFU activity in around 30 s with an LOD of 1.0 × 10^−5^ M of 2-chloro-4-NP. This biosensor method also had good sensitivity and was selective for AFU [115]. A fluorescence biosensor detected AFU activity with excellent selectivity and sensitivity in a different study. The fluorescence biosensor detected AFU activity based on the fluorescence quenching of CdTe semiconductor quantum dots. The AFU detection limit using the biosensor was 0.01 U/L, which provided a fast and sensitive detection mechanism for AFU in HCC [116]. A different fluorescence quenching biosensor method has also shown remarkable potential for detecting AFU. The biosensor detected AFU based on the fluorescence quenching of carbon dots (C-dots) and AuNPs. An LOD of 3.4 nM was achieved using the biosensor, providing another method of detection with high selectivity and sensitivity [117].

### 4.2. Glypican-3 (GPC-3)

Glypican-3 (GPC-3) is a membrane-bound heparan sulfate proteoglycan. GPC-3 is typically expressed in the fetal liver, but in HCC, it was found to be aberrantly expressed. The proteoglycan was found to play a role in the pathogenesis of HCC by influencing signaling pathways such as the Wnt proteins leading to hepatocyte oncogenesis [118,119]. The diagnostic potential of GPC-3 in detecting HCC has significantly risen over recent years. The sensitivity and specificity of GPC-3 as a biomarker for HCC were 69 and 93%, respectively, making it a specific biomarker for HCC [120].

Biosensors are being used to detect GPC-3 in HCC and are showing remarkable results. Cyclic voltammetry and electrochemical impedance spectroscopy were used to detect GPC-3 and AFP, targeted by antibody-coupled magnetite nanoparticles decorated with hyperbranched, amino-functionalized dendrimers. The LOD of this methodology was 70 and 50 pg/mL for AFP and GPC-3, respectively. This biosensor provides a fast and sensitive method for detecting GPC-3 in HCC [121]. The FRET-based aptasensing strategy was able to detect GPC-3 with high specificity. The aptasensor, constructed from gold carbon dots and magnetic graphene oxide nanosheets, can specifically detect GPC-3. In this sensing method, a gold carbon-dot-labeled GPC-3 aptamer was adsorbed on magnetic GO nanosheets, and the fluorescence of QDs was quenched due to FRET from QDs to nanosheets. In the presence of the GPC-3 target, the aptamer detaches from the nanosheet and recovers the fluorescence signal. The fluorescent aptasensor had an LOD of 3.01 ng/mL, showing significant potential applications in detecting GPC-3 in HCC [122]. Furthermore, many studies have explored the use of electrochemical aptasensors for detecting GPC-3 in HCC. A study demonstrated using an electrochemical aptasensor for detecting GPC-3 by combining hemin-reduced graphene oxide-platinum nanoparticles with reduced graphene oxide–gold nanoparticles. The sensitivity of the aptasensor was high, with an LOD of 0.001 μg/mL, holding great promise for its role in diagnosing HCC [123]. An electrochemical aptasensor was developed in another study to detect GPC-3 using platinum@palladium nanoparticles decorated with hemin-reduced graphene oxide. The GPC-3 aptasensor offered a high sensitivity and selectivity, with an LOD of 0.181 ng/mL and a sensitivity of 0.0446 μA μM/cm^2^, providing a promising method for detecting GPC-3 in HCC [124]. Another study developed an electrochemical aptasensor using reduced graphene oxide–hemin nanocomposites to detect GPC-3. The nanocomposites were modified on a screen-printed electrode surface and had a sensitivity of 0.134 μA/μM/cm^2^ with an LOD of 2.86 ng/mL, exhibiting excellent sensitivity in the detection of GPC-3 in HCC [125]. Moreover, GPC-3 detection with an electrochemical aptamer nano-biosensor had high sensitivity and selectivity. The nano-biosensor was based on hemin/graphene nanohybrid peroxidase-like catalytic silver deposition to detect GPC-3. The nano-biosensor had an LOD of 3.16 μg/mL and a sensitivity of 0.807 μA/μM/cm^2^, providing another sensitive method of detecting GPC-3 in HCC [126]. In the preceding year, Li et al. devised a highly sensitive homogeneous aptasensor for detecting Glypican-3 (GPC3) with enhanced accuracy and a reduced detection limit [3]. This innovative system is based on fluorescence resonance energy transfer (FRET), where gold carbon dots labeled with the GPC3 aptamer (AuCDs-GPC3Apt) serve as the donor, and magnetic graphene oxide (Fe_3_O_4_/GO) nanosheets act as the acceptor. The synthesis of AuCDs was accomplished through a one-step hydrothermal method, ensuring ample fluorescence for the system. The FRET phenomenon between AuCDs-GPC3Apt and Fe_3_O_4_/GO resulted in a reduction in the overall fluorescence intensity. Upon introducing the target GPC3 to the FRET system, the fluorescent AuCDs-GPC3Apt were bound explicitly to GPC3, inducing a folded structure that separated AuCDs-GPC3Apt from Fe_3_O_4_/GO nanosheets. The magnetic separation of Fe_3_O_4_/GO facilitated the restoration of fluorescence in free-labeled AuCDs-GPC3Apt. The LOD for GPC3 in this system was determined to be 3.01 ng/mL, with a linear detection range from 5 to 100 ng/mL.

Li et al. devised an electrochemical aptasensor for the detection of GPC-3. The sensor utilized a construct comprising reduced graphene oxide-carboxymethyl chitosan-hemin-palladium nanoparticles (RGO-CMCS-hemin/Pd NPs) in conjunction with the GPC3 aptamer (GPC3apt), enabling GPC3 identification and analysis [127]. The RGO-CMCS-hemin/Pd NPs demonstrated remarkable biocompatibility, a large specific surface area, and robust electrical conductivity. The GPC3apt, upon interaction with GPC3, formed a GPC3-aptamer conjugation, inducing changes in the electron transfer impedance and corresponding electrical signals. This aptasensor exhibited a commendable LOD of 1.0 ng/mL and a linear detection range spanning from 1.0 to 100.0 ng/mL, showcasing its potential for precise GPC-3 detection and analysis in relevant clinical scenarios.

Chen et al. designed a label-free electrochemical aptasensor to detect GPC3 [128]. The sensor had a reduced graphene oxide–hemin–chitosan (RGO–H–CS) nanocomposite-modified screen-printed electrode (SPE) as the biosensing platform, and the GPC3 aptamer served as the recognition element. In the presence of GPC3, the aptamer is specifically bound to the target GPC3, forming GPC3-aptamer conjugations on the sensing surface. This interaction changed the electrochemical redox signal of hemin (Fe(III)/hemin(Fe(II))) within the RGO–H–CS nanocomposite, which was recorded by DPV. The aptasensor demonstrated a noteworthy LOD of 7.9 ng/mL and a linear detection range from 0.01 µg/mL to 10.0 µg/mL.

Shi et al. engineered a GPC-3 detection sensor by fabricating a biosensing platform with reduced graphene oxide-chitosan-ferrocene and the deposition of Pt–Pd bimetallic nanoparticles (RGO-CS-Fc/Pt–Pd BNPs) [129]. The recognition element utilized in this sensor was the GPC3 aptamer (GPC3apt). The GPC3apt selectively interacted with GPC3 in the test samples, forming GPC3-aptamer complexes on the biosensing platform. This interaction amplified the electron transfer impedance and reduced the redox current of ferrocene. The sensor exhibited a remarkable LOD of 3.67 ng/mL and a linear detection range from 0.001 to 10 µg/mL. There are several biosensors constructed for the detection of GPC3 including electrochemical, fluorescence, and FRET. Aptamers are frequently used as bioreceptors and nanomaterials such as metal oxides and GO are used to improve the performance of the sensor. Among all types of sensors, electrochemical biosensors perform well.

### 4.3. Alpha-Fetoprotein (AFP)

AFP, which acts as a transporter of ligands such as bilirubin and fatty acids, is the most common serologic marker of HCC. In a healthy individual, it is only secreted in the fetal liver throughout development before dropping and remaining low for a person’s lifespan [130]. In HCC, however, AFP tends to be aberrantly overexpressed. It is linked to a more aggressive tumor and a worse prognosis overall. Although 30% of patients remain AFP-negative, it remains a handy screening tool for HCC [10,131].

Zhou et al. developed an aptasensor based on Forster resonance energy transfer (FRET) [132]. AFP-aptamer-labeled luminescent CdTe quantum dots (QDs) act as a donor, while anti-AFP antibody functional gold nanoparticles (AuNPs) act as the acceptor. Once AFP is added, the affinity between AFP, its aptamer, and the antibody brings the QDs and AuNPs closer, which quenches the fluorescence of the QDs by FRET. This simple and reliable aptasensor showed an LOD of 400 pg/mL. Li et al. created an aptasensor using mismatched catalytic hairpin assembly (MCHA). First, the trigger is locked by an aptamer, and then AFP is introduced. The aptamer then combines with the AFP, releasing the trigger and initiating the MCHA cycle. This forms a double chain complex (H1@H2), along with a double chain structure containing the fluorophore and BHQ1 (its quenched group). It initiates a displacement reaction leading to fluorescence recovery. This fast and reliable sensor could detect down to an LOD of 0.033 ng/mL within 60 min [133]

**Figure 6:**
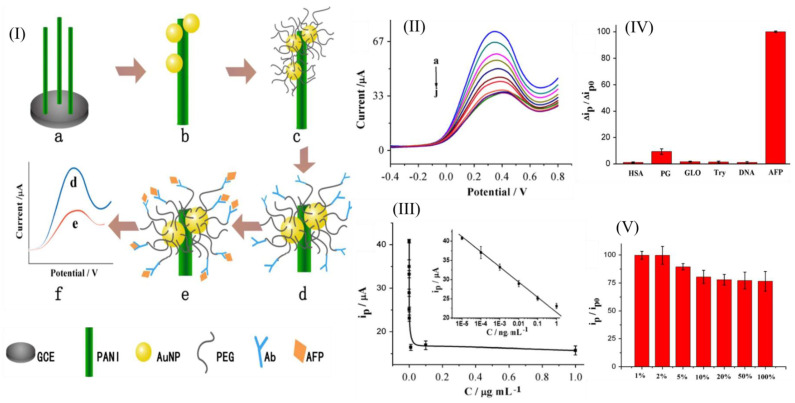
(**I**) Illustration of AFP immunosensor. (a) PANI nanowires deposited on the GCE, (b) AuNPs electrodeposition, (c) PEG modification, (d) AFP antibody immobilization, (e) AFP target capturing and (f) DPV current signal recording. (**II**) DVP signal with increasing concentration of AFP. (**III**) Dynamic signal variation with AFP concentrations. (**IV**) Cross reactivity with other biomolecules. (**V**) Antifouling property of the AFP immunosensor in human serum [136] we upwith copyright permission.

Zhou et al. developed a graphene-based nanocomposite biosensor to detect AFP [134]. They used a nanocomposite made from gold nanoparticles and dextran-reduced graphene oxide (AuNPs-Dex-RGO). They then loaded it onto a glassy carbon electrode (GCE) before incubating it with an AFP antibody (Ab). The resulting Ab/AuNPs-Dex-RGO/GCE electrode could detect AFP at concentrations as low as 0.05 pg/mL. Hui et al. developed a different biosensor using polyaniline (PANI) nanowires [135] (Figure 6). These were functionalized using AuNPs and polyethylene glycols (PEGs). An antibody was then immobilized on the PEG/AuNP/PANI composite. This label-free, reagent-less immunosensor utilizes the redox current of PANI as a sensing signal to detect AFP at a low LOD of 0.007 pg/mL. In another study, Shan et al. [136] developed a novel nanocomposite biosensor using poly(methylene blue)-Au. The Ab was immobilized onto the nanocomposite—for signal amplification—which formed the probe. The substrate was a graphene-Au-modified electrode. Using this system, they achieved an LOD of 19.6 fg/mL. In a different study, an immunoassay that used AgNPs-Ab probes was fabricated [137]. This electrochemical biosensor uses the interactions between the probes and AFP to induce the aggregation of AgNPs, increasing their size and reducing their concentration. Then, they measured the transduction signals using NIE, which further amplified their sensitivity. This sensor enabled an LOD of 5 pg/mL. In a similar study, an AgNP-linked immunoassay was developed utilizing aggregation-induced emission [138]. The sensor utilizes the dissolution of AgNP probes into Ag^+^ to activate a fluorogenic Ag probe by tetrazolate-Ag^+^ complexation. A photoelectrochemical biosensor was developed by Li et al. [139]. It uses photoelectric-active substances as a signal transducer. Light excites the photosensitive substance, and the electrical signal is measured. Au/Cs_x_WO_3_ heterogeneous films were prepared and used to enhance the electrode. This specific, stable biosensor was able to achieve an LOD of 7 pg/mL. Yao et al. developed a GMR-based biosensor to detect AFP rapidly [140]. GMR (giant magnetoresistive effect) sensors use a magnetically labeled biomolecule which generates a magnetic field that impacts the sensor resistance upon binding with the target and the captured antibody in a sandwich format. Using this sensor, they could detect AFP in concentrations as low as 10 ng/mL. These biosensor systems offer rapid, convenient, and cheap strategies to detect AFP sensitively.

### 4.4. Golgi Protein 73 (GP73)

GP73 is a glycoprotein, expressed on the surface of the Golgi apparatus, that is typically expressed in biliary epithelial cells but can rarely be expressed in hepatocytes. However, in cases of HCC, GP73 expression tends to be elevated [141]. It has been reported to be a potential marker for cirrhosis and HCC, although it predominantly rises in cases of cirrhosis [136].

For the sensitive detection of GP73, Yu et al. developed an electrochemical immunosensor that can directly detect the glycoprotein from serum [142]. They used CdSe quantum dots, modified by aleuria aurantia lectin (AAL) and PEG, as a substrate. A glassy carbon electrode, modified with electrochemically reduced graphene oxide, was given the anti-GP73 antibody. This system enabled the detection of GP73 at an LOD of 12 pM.

In another study, Liang et al. developed an aptasensor using graphene QDs and reduced graphene oxide nanosheets [143]. Molybdenum disulfide @ reduced graphene oxide (MoS@RGO) nanosheets were used as fluorescence receptors, while the QDs, doped with nitrogen and labeled with GP73 aptamer, were used as a probe. Typically, the nanosheets quench the fluorescence of the QDs via FRET. When GP73 is added, the QDs bind to GP73, pushing them away from the nanosheets. This inhibits the FRET process, resulting in fluorescence. With this biosensor, GP73 could be detected in quantities as low as 4.54 ng/mL. Another fluorescence biosensor was developed by Liu et al. [144]. Mn-modified CdTe/CdS QDs were synthesized and capped with anti-GP73 antibodies before conjugating with protein A/G agarose beads. These could capture GP73 and be separated by a centrifuge, leading to the quenching of their fluorescence. With this sensor, GP73 could be detected at an LOD of 10 ng/mL. A different biosensor based on reduced graphene oxide-carboxymethyl chitosan-hemin/platinum@palladium nanoparticles (RGO-CMCS-hemin/Pt@Pd NPs) was developed by Li et al. [145]. After synthesizing the NPs, aminylated GP73 aptamers were bound to it to form the recognition probe. In the presence of GP73, the NPs form a sandwich structure with GP73 and a second, unmodified aptamer (the capture probe). In the presence of H_2_O_2_, this oxidizes 3,3′,5,5′-tetramethylbendzidine (TMB), which can be detected by UV absorption. This sensor achieved an LOD of 4.7 ng/mL.

A ratiometric immunosensor based on electrochemiluminescence was synthesized to detect GP73 [146]. A DNA tetrahedron nanostructure (DTN) was used as a self-assembly platform. The Au electrode was treated and then immersed in the DTNs. Then, MCH, methylene blue, streptavidin (SA), and anti-GP73 Ab were added. The probe was immersed in GP73 and then into a solution of Ab labeled with tris(4,4′-dicarboxylicacid-2,2′-bipyridyl)ruthenium(II) dichloride (the working signal) for 1 h to form an antibody–antigen–antibody sandwich. The ECL signal from the labeled antibodies and the electrochemical signal of methylene blue were measured, and the concentration of GP73 could be quantified based on the ratio. With this system, the detection of GP73 could be achieved at an LOD of 15 pg/mL.

Lin et al. studied utilizing DNA tetrahedron nanostructures (DTNs) for their simple synthesis, high yield, structural stability, and mechanical rigidity. The three-dimensional architecture of DTNs was particularly advantageous, as it provided an effective biosensing interface, enhancing the binding efficiency between antigenic proteins and antibodies. In this research, the electrochemiluminescence (ECL) reagent, specifically tris(4,4′-dicarboxylicacid-2,2′-bipyridyl)ruthenium(II) dichloride [Ru(dcbpy)_3_]Cl_2_, was introduced to the electrode by forming the classical sandwich complex of the antibody–antigen–antibody. The ECL response demonstrated a direct correlation with the concentration of the target molecule, Golgi protein 73 (GP73), thereby establishing a highly selective assay [147]. This culminated in developing a ratiometric immunosensor based on the ratio of ECL emitted by Ru(dcbpy)3Cl_2_. The achieved LOD for GP73 was an impressive 15 pg/mL. Lin et al.’s work holds significant promise for applications in biosensing and immunodiagnostics.

Sun et al. engineered an aptasensor featuring nitrogen-doped graphene quantum dots (NGQDs) labeled with GP73 aptamer (GP73Apt) as a fluorescence probe [148]. The complementary component in this innovative setup was molybdenum disulfide @ reduced graphene oxide (MoS_2_@RGO) nanosheets, serving as the fluorescent receptors. MoS_2_@RGO nanosheets effectively quenched the fluorescence of NGQDs-GP73Apt through fluorescence resonance energy transfer (FRET) mechanisms. In the presence of GP73, NGQDs-GP73Apt are selectively bound to GP73, forming deployable structures that spatially separate NGQDs-GP73Apt from MoS_2_@RGO nanosheets. This structural change obstructed the FRET process, leading to the fluorescence recovery of NGQDs-GP73Apt. The LOD for GP73 in this system was determined to be 4.54 ng/mL, with a linear detection range spanning from 2.5 ng/mL to 100 ng/mL.

Cancer diagnosis in the early stages requires selective and sensitive sensors capable of detecting specific biomarkers. To tackle this problem, Li et al. developed a dual-signal sandwich-type electrochemical aptasensor to determine GP73 [148]. This aptasensor utilized hemin-reduced graphene oxide–manganese oxide (H-rGO-Mn_3_O_4_) nanozymes as the basis. Gold@poly(o-phenylenediamine) (Au@POPD) nanohybrids, known for their large specific surface area and conductance, were co-electrodeposited onto a screen-printed electrode (SPE) surface to immobilize the GP73. The sensor demonstrated an LOD of 0.0071 ng/mL and a linear detection range from 0.01 to 100.0 ng/mL.

Zhang et al. pioneered the development of a highly sensitive electrochemical immunosensor, integrating the precision of the proximity ligation assay (PLA) with the efficiency of enzyme-powered recycling amplification for the determination of GP73 [149]. The selective PLA is initiated by the affinity binding of two labeled antibody–DNA complexes (P1-RAb and P2-MAb) to the target protein, enhancing specificity. The resulting immunocomplex engages with DNA2, facilitating the release of methylene blue-labeled mononucleotide fragments through the action of exonuclease III, driving the recycling amplification process. The liberated fragments quickly diffuse to the surface of the nafion-modified indium tin oxide electrode, generating a robust electrochemical signal. This innovative immunosensor for GP73 detection achieves an impressive LOD of 0.10 pg/mL, with a linear detection range spanning from 0.3 pg/mL to 6.0 ng/mL.

Wei et al. devised an automated methodology employing the ACL2800 automatic chemiluminescent analyzer, in which magnetic nanoparticles (MNPs) and chemiluminescence played integral roles [150]. This approach hinged on a sandwich strategy, orchestrating interactions between a biotin-labeled capture monoclonal antibody, the target GP73, and an acridinium ester (AE)-labeled reporter monoclonal antibody. Additional components encompassed the binding of streptavidin-labeled MNPs to the biotin-labeled capture monoclonal antibody, culminating in the chemiluminescent detection of AE-linked targets. The LOD for GP73 was determined to be 1.19 ng/mL. GP73 biomarker detection from different types of biosensors have been reported. In most cases, electrochemical techniques integrated with various types of nanomaterials, quantum dots, and antibodies have used to enhance the sensitivity. The hemin-reduced graphene oxide–manganese oxide (H-rGO-Mn_3_O_4_)-based aptasensor showed a high LOD of 0.0071 ng/mL compared to other GP73 biosensors [148]

### 4.5. Osteopontin (OPN)

OPN is an integrin-binding glycophosphoprotein that plays a role in many different cancers, aiding in migration, invasion, and metastasis [151]. It is usually not expressed in hepatocytes, and an elevated expression of OPN is linked to HCC. It was shown to be more sensitive to HCC than AFP, and when combined with AFP, it may reach a sensitivity and specificity rate for HCC of 95% and 96%, respectively [152]. Thus, OPN is a strong potential biomarker for HCC.

Numerous aptasensors have been synthesized to detect OPN. Makuma et al. developed a lateral flow aptasensor to detect OPN [153]. AuNP-SA conjugates were prepared and dispensed on the conjugate pad. Anti-OPN Abs were adhered to the test zone of the nitrocellulose membrane. OPN was incubated with a biotinylated aptamer, forming an aptamer–OPN complex. These were captured by the AuNP-SA complex on the conjugate pad, and, as they flowed across the strip, captured by the anti-OPN antibody. The unbound aptamer moved on and was bound by an ssDNA probe in the strip’s control zone. This device could detect OPN within 5 min at a 0.1 ng/mL LOD. Meirinho et al. also developed a voltametric aptasensor to detect OPN [154]. An RNA aptamer was synthesized and immobilized onto a streptavidin-modified gold electrode surface of a strip containing a silver pseudo-reference electrode and a gold counter-electrode. A 5 mM solution of [Fe(CN)_6_]^3−/4−^ was used as a redox probe. For detection, the sample was dropped onto the electrode, incubated, and washed, and then the redox probe was dripped onto the electrodes until all three were immersed. Then, a CV assay was performed, and the results correlated with the concentration of OPN. This device could detect OPN down to an LOD of 520 ng/mL (3.7 nM). In another study, Meirinho et al. developed an electrochemical aptasensor by selecting a DNA aptamer using SELEX [155]. They first prepared an aptamer using the SELEX selection process. A similar aptasensor to the one prior was then constructed, with the aptasensor immobilized onto the gold electrode surface, and the same process to measure the OPN was performed. This device achieved an LOD of 1.3 ± 0.1 nM. Zhou et al. constructed an impedimetric bioassay—to detect OPN—using zirconium oxide NPs @ graphene-like nanofiber (ZrO_2_@GNF) [156]. ZrO_2_@GNF nanohybrids, developed from zirconium-based metal–organic frameworks (UiO-66), were entrapped in electrospun polyacrylonitrile (PAN) fibers (denoted UiO-66@PAN). The structure produced—ZrO2@GNF—had both enhanced conductivity and improved bioaffinity towards OPN aptamer strands. The biosensor was reliable and stable, showing a final LOD of 4.76 ng/mL. Chen et al. prepared an aptasensor based on iron oxide NPs (IONPs) [157]. These were modified by (3-aminopropyl) triethoxysilane (APTES) to attach them to the plate’s surface. Then, an antibody was added to the well. APTES-IONP were added onto the well, followed by a washing step. Then, an anti-OPN Ab was added, and different concentrations of OPN were added to the antibody to modify it well, followed by a masking step. This was followed by the addition of the biotinylated aptamer and streptavidin HRP to delineate the interaction of the antibody–OPN–aptamer sandwich. Quantitative analysis was performed by measuring absorbance.

Sharma et al. developed a label-free transparent immunosensor based on single-walled carbon nanotubes (SWCNTs) to detect OPN [158]. COOH functionalized the SWCNTs. Then, dielectrophoresis allowed their deposition between two gold/indium tin oxide electrodes. Anti-OPN Abs were then adhered to the SWCNTs. After adding OPN, the relative resistance change was measured, which can be used to determine OPN concentration. The LOD for this device was found to be 0.3 pg/mL.

Zhou et al. developed an electrochemical aptasensor for the detection of OPN utilizing a nanohybrid composed of Ti_3_C_2_T_x_ MXene and phosphomolybdic acid (PMo12) were embedded with polypyrrole (referred to as PPy@ Ti_3_C_2_T_x_ /PMo12) [159]. This nanomaterial served as an effective platform for robustly immobilizing the OPN aptamer, enabling the construction of an aptamer biosensor. The sensor exhibited a low LOD at 0.98 fg/mL and a linear detection range spanning from 0.05 pg/mL to 10.0 ng/mL. In the subsequent year after Zhou et al.’s study, the same researcher discovered another method for detecting osteopontin (OPN) utilizing a nanohybrid composed of zirconium oxide nanoparticles and graphene-like nanofiber (referred to as ZrO_2_@GNF) [160]. This approach demonstrated a low LOD at 4.76 fg/mL and a linear detection range spanning from 0.01 pg/mL to 2.0 ng/mL. Aptamers and antibodies in combination with nanomaterials were used for biosensor construction. The LOD of the sensors had low pg/mL levels obtained from electrochemical methodologies

### 4.6. Squamous Cell Carcinoma Antigen (SCCA)

Squamous cell carcinoma antigen (SCCA) belongs to the high-molecular-weight serine protease inhibitors (serpins) group and is found in stratified squamous epithelia’s upper layers. SCCA, the acidic isoform, can be identified in many epithelial malignancies, such as cervical, lung, head, and neck cancers. SCCA is not often found in a healthy liver. However, its abnormal expression has recently been observed in HCC at both the molecular and protein levels [158,161]. An innovative and highly sensitive electrochemical immunosensor, designed as a sandwich structure, was created using CD-GN as a base and Pt/PdCu-3DGF as markers. This sensor achieved the adequate amplification of numerous signals. The CD-GN, which has been modified on the immunosensor surface, possesses a specific surface area that enables it to collect more antibodies through host–guest interaction. The Pt/PdCu-3DGF material can efficiently bind detection antibodies through Pd-NH_2_ and Pt-NH_2_, demonstrating exceptional electrochemical catalytic activity for reducing H_2_O_2_. The immunosensor that was created demonstrated a sensitive response to SCCA under ideal circumstances, with two distinct linear ranges. The linear ranges for measurement are 0.0001–1 ng/mL and 1–30 ng/mL, and the LOD is 25 fg/mL.

Furthermore, the suggested immunosensor exhibited commendable reproducibility and stability [162]. Nevertheless, in real life, the SCCA level in healthy persons’ blood serum is often below 1.5 ng/mL, posing a significant obstacle to the identification and precise measurement of SCCA. To address the drawbacks associated with the intricate procedure and expensive nature of earlier detection approaches, Qiu et al. developed a PEC immunosensor platform utilizing Au-NPs@Zn-MOF nanocomposite materials. This work validated that the Au-NPs@Zn-MOF-based PEC immunosensor exhibited good selectivity, sensitivity, and stability for SCCA detection, surpassing the previously described PEC immunosensor. The change in photocurrent associated with the specific detection of SCCA exhibited a linear correlation with the logarithm of SCCA concentration throughout the range of 5.0 pg/mL to 15.0 ng/mL. The LOD was 2.34 pg/mL. The PEC immunosensor exhibited exceptional selectivity, sensitivity, stability, and reproducibility for detecting SCCA. However, the process of creating gold nanocomposites remains intricate, requiring significant time and manual effort. Therefore, the immunosensor encountered notable obstacles and opportunities for enhancement in the prompt identification of tumor markers [163].

A novel biosensor was designed to overcome standard testing methodologies’ limitations, such as the need for labeling, the long assay time, and the low sensitivity and selectivity. The biosensor utilizes a triangular array of silver nanoparticles to detect SCCA using Localized Surface Plasmon Resonance (LSPR). The chip is immobilized with monoclonal anti-SCCA antibodies for direct detection. The ultrasensitive and specific LSPR system effectively evaluated various concentrations of SCCA in both buffer and human serum. The technology demonstrated a linear quantitative detection range of 0.1–1000 pM under ideal conditions [164].

Furthermore, Fan et al. devised an innovative immunosensor platform that does not require labels. This platform is built on CdS-sensitized Fe-TiO_2_ nanocomposites and can detect SCCA with exceptional sensitivity. The biosensor exhibited a broad detection range of 0.001 ng/mL to 75 ng/mL and a low LOD of 0.22 pg/mL due to the favorable photoelectrochemical (PEC) response of CdS-enhanced Fe-TiO_2_ [165]. Later, in 2019, Fan et al. created a photoelectrochemical immunosensor that uses BiOBr/Bi_2_O_3_ heterostructures and visible light to detect SCCA with high sensitivity. Through the self-sacrificial synthesis method, the interaction between BiOBr and S_2_ resulted in the formation of Bi_2_S_3_ on the surface of BiOBr microspheres. This process led to the development of remarkable photoelectrochemical activity under visible light. Dopamine undergoes self-polymerization to generate a polydopamine layer on its surface, enhancing the durability of photoelectric signals and facilitating antibody binding. The intensity exhibited a linear decline when the SCCA concentration’s logarithm varied throughout the 0.001–75 ng/mL range. The detection limit was determined to be 0.3 pg/mL [166].

### 4.7. Hedgehog (Hh) Ligands

Hedgehog (Hh) molecules have a crucial role as soluble factors in controlling cell proliferation and differentiation in various stages of development, maintaining the balance of adult tissues, and influencing cancer development. Three kinds of Hh ligands, namely Sonic hedgehog (SHh), Indian hedgehog, and Desert hedgehog, have been linked to this pathway in humans [167]. The elevated expression of SHh is strongly associated with the spread of cancer to other parts of the body, resistance to drugs, and a poor prognosis for patients with HCC [168]. An aptasensor-based assay was effectively created by linking Texas-Red-labeled AP32 to microbeads and was utilized to examine SHh concentration in hepatoma cell lysates, serum, and HCC tissues. The approach demonstrated a wide range of detection capabilities, spanning from 0.07 to 62.5 nM, with a very sensitive LOD of 69 pM.

Additionally, it achieved a recovery rate of 104.6 ± 3.9% when applied to blood samples. When the assay was employed to quantify the SHh concentration in tissue lysates, the findings revealed a positivity rate of 57.1% and a specificity of 100% in differentiating 28 HCC specimens from normal tissues. The assay proved effective in detecting HCC in situations where AFP was negative [168]. In another study, a high-sensitivity electrochemical biosensor for the SHh assay was developed by utilizing a novel aptamer specific to SHh and the combination of the primer exchange reaction (PER) and catalytic hairpin assembly (CHA). SHh and its aptamer combine to generate a strand that can undergo hybridization with the dumbbell-shaped hairpin, thereby inducing PER and the production of many new ssDNAs. In order to further facilitate the confinement of numerous methylene blue (MB)-labeled sequences on the sensor surface, these strands initiate CHA cycles. The amplified assay of SHh with an LOD of 4.1 pM is made possible by the significantly magnified electrochemical current signals of the surface-attached MB labels, resulting from the two-step cyclic amplification of PER and CHA [169].

Recent research has demonstrated that tissue remodeling is crucial for the progression of hepatocellular carcinoma. As a serine protease, Kallikrein 6 (KLK6) is implicated in the tissue remodeling process that governs the formation of HCC. For clinical KLK6 detection, a surface-confined peptide network that can display distinct structural characteristics upon protease cleavage and electrochemical treatment is devised as a highly sensitive biosensor. In the presence or absence of the protease, the network exhibits a unique behavior characterized by a prominent signal response and a low background, which results in an LOD of less than 1 pM and an adequate dynamic range [170].

### 4.8. β-Catenin

The Wnt signaling pathway, primarily comprising the intracellular effector β-catenin, is significant in the development and metastasis of HCC, among the numerous pro-metastatic pathways recently identified and characterized [171]. One study presented a method for quantifying the involvement of β-catenin in pro-metastatic cell signaling. The LOD was found to be less than 10 pM, which was calculated using a signal-to-noise ratio of 3:1. Given these circumstances, two probes based on peptides are developed; these probes are derived from the molecular partners of β-catenin that participate in these protein interactions. Additionally, movable groups are intentionally integrated into the probes to facilitate a catalytic cross-linking between the two probes under electrochemical control [172].

### 4.9. Lipocalin-2 (LCN2)

LCN2, also known as Lipocalin-2, is a glycoprotein secreted and stored in human neutrophils. LCN2 is primarily involved in the transportation of tiny ligands, which are known to play a role in inflammation, iron metabolism, and the initiation of apoptosis [173,174]. A recent HCC-microarray analysis study revealed a significant increase in the expression of LCN2, indicating its potential as a quantitative biomarker [175]. The diagnostic approach described is based on a single-stranded DNA aptamer and can detect LCN2, a biomarker in the serum of patients with HCC. The method uses a sandwich assay format with an LOD of 0.6 ngmL^−1^ [176].

### 4.10. Pentraxin-3 (PTX-3)

Pentraxin-3, also known as tumor necrosis factor-stimulated gene 14 (TSG-14), is a long pentraxin of the pentraxin superfamily. Structurally, PTX3 has an unrelated N-terminal domain linked to a pentraxin-like C-terminal domain. PTX-3 acts as a linker between innate immunity, inflammation, tissue repair, and cancer. PTX-3 expression levels are related to the prognosis in certain cancers, including HCC [177]. Zhang et al. developed a biosensor for Pentraxin-3 (PTX-3) detection, employing nanocomposites of functionalized multi-walled carbon nanotubes (MWCNTs) and gold nanowires (AuNWs) [178]. The PTX-3 biosensor, characterized by excellent electrochemical activity and biocompatibility due to the inclusion of carbon and metal nanomaterials, demonstrated heightened performance with the synergistic combination of MWCNTs and AuNWs. This sensor achieved a remarkable LOD of 0.16 pg/mL and a linear detection range from 0.001 ng/mL to 1000 ng/mL. The innovative design allows for highly sensitive and precise PTX-3 detection in human serum, facilitated by a simple and effective non-enzymatic reaction.

## 5. Other Biomarkers

There are many other biomarkers used for the diagnosis of diseases including whole cells, pathogenic microbes, parasites, etc. For example, a simple hydrophobic attraction of a pathogen from the biological sample techniques was used for concentrating the target pathogen for an efficient analysis [179]. Hu et al. devised an electrochemical aptasensor tailored for detecting glycoprotein biomarkers [180]. This sensor employed a DNA-aptamer-modified electrode as the sensing interface, providing specificity for the targeted glycoprotein tumor biomarkers. The methodology involved attaching alkyl-halide initiators for atom transfer radical polymerization (ATRP) to the captured biomarkers via esterification crosslinking between the boronic acid group and the cis-dihydroxyl sites of the conjugated oligosaccharide chains. Subsequently, long-chain polymers were grown through electrochemically controlled ATRP (eATRP), facilitating the efficient recruitment of ferrocene detection tags. This electrochemical aptasensor demonstrated an impressively low LOD of 0.32 pg/mL, showcasing its potential for the susceptible detection of glycoprotein tumor biomarkers in a quantitative range relevant to biomedical applications.

A significant development emerged by introducing the ECL acceptor probe, Pd NPs@NH_2_-MIL-53, onto the modified glassy carbon electrode (GCE) through specific recognition mechanisms. This interaction resulted in a marked reduction in the ECL signal originating from g-C_3_N_4_@Au NPs. The research achieved a strikingly low LOD of 3.4 fg/mL for amyloid-beta protein [181]. The findings of this study not only underscore the potential for highly sensitive biomarker detection but also hold substantial promise for diverse applications in the domains of diagnostics and research.

Al Shami et al. engineered an electrochemical immuno-sensor to detect the biomarker Midkine [182]. The sensor’s functionality relied on the covalent immobilization of Anti-Midkine antibodies using carbodiimide chemistry. This process occurred on carbon screen-printed electrodes modified with carboxylated multi-walled carbon nanotubes. Characterizing the sensor’s development involved techniques such as cyclic voltammetry, electrochemical impedimetric spectroscopy, Fourier transform infrared spectroscopy, and atomic force microscopy. The sensor demonstrated an impressive LOD at 0.8 pg/mL, and its linear detection range spanned from 1 pg/mL to 100 ng/mL.

Aflatoxin B1 (AFB1) is a prevalent and well-identified toxin strongly linked to the development of HCC [183]. An electrochemical impedance spectroscopy approach has been devised to determine AFB1 using MWCNTs/RTIL-composite-film-based immunosensors. The method is both sensitive and convenient. The calibration curve for AFB1 exhibited linearity within the range of 0.1–10 ng mL, with an LOD of 0.03 ng mL [184].

Damiati et al. developed a mass-based piezoelectric sensor for detecting whole cells, specifically liver cancer cells (HepG2), utilizing antibodies [185]. This study introduces an effective acoustic and hybrid three-dimensionally (3D) printed electrochemical biosensor for liver cancer cell detection. The biosensors operate by recognizing the highly expressed tumor marker CD133 on the surface of liver cancer cells. Detection is facilitated by recrystallizing a recombinant S-layer fusion protein (rSbpA/ZZ) onto the sensor surface. The fused ZZ-domain allows for the precise immobilization of anti-CD133 antibodies. These readily accessible antibodies serve as a sensing layer, enabling the efficient detection of liver cancer cells (HepG2). The recognition of HepG2 cells was investigated in situ using a quartz crystal microbalance with dissipation monitoring (QCM-D), allowing the label-free, real-time detection of living cells on the modified sensor surface under controlled conditions. The device has an LOD of 1 × 10^3^ cells/mL and a linear detection range spanning from 1 × 10^5^ cells/mL to 3 × 10^6^ cells/mL.

Chen et al. developed a sensor for the label-free detection of whole cells, specifically liver cancer cells (HepG2), based on a cantilever [186]. The sensor utilized an aptamer to detect HepG2 cells. This study presented a microcantilever array aptasensor for detecting HepG2 cells without labels. The sensing microcantilevers were functionalized with HepG2-cell-specific aptamers. The reference microcantilevers were modified with 6-mercapto-1-hexanol self-assembled monolayers to eliminate interferences from the environment. The sensor demonstrated an LOD of 300 cells/mL, indicating its capability to detect low concentrations of HepG2 cells. Furthermore, the linear range of detection extended from 1 × 10^3^ to 1 × 10^5^ cells/mL, providing a broad dynamic range for accurate and reliable measurements across different concentrations. Biosensors developed hepatocellular carcinoma-specific biomarkers using different biosensing techniques have summarized in Table 2. 

**Table 2:** Summary of linear range and detection limits of hepatocellular carcinoma-specific biomarkers using different biosensing methods.

Target Biomarker	Materials Used for Detection	Recognition Element	Readout	Linear Range	LOD	References
**Nucleic Acid Biomarkers**						
Tumor Protein (TP53) gene	Sulfhydryl-ended hairpin DNA probes tagged with methylene blue, gold electrodes	Complementary ssDNA probe	DPV	10–1000 nM	10 nM	[43]
	Electrochemical growth of AuNps on aligned multi-walled carbon nanotubes (A-MWCNTs)	Complementary ssDNA probe	EIS	10^−7^–10^−15^ M	1.0 × 10^−17^ M	[48]
miR let-7a	Silver nanoparticles (AgNPs), AgNPs-extracted propolis (bee glue)-modified carbon paste electrode (APCPE)	Complementary ssDNA probe	SWV and EIS	10^−3^ fM–1 µM	10^−3^ fM	[80]
miR-122	2-aminopurine (2-AP)-labeled stem-loop and thioflavin T (ThT)-induced G-quadruplex-forming sequence	2-AP-labeled target complementary stem-loop hairpin DNA	Ratiometric fluorescence	0.5–50 nM	72 pM	[81]
miR-122 and AFP	dsDNA duplex generated from miRNA complementary and AFP aptamer and methyl violet intercalated in dsDNA duplex	AFP aptamer and miRNA Complementary DNA	RLS	5–100 μg/L (mir122) 200 pM–10 nM (AFP)	98 pM (mir) 0.94 μg/L (AFP)	[187]
miR-122	dsDNA duplex generated from miRNA complementary and G-quadruplex-forming sequences	miRNA Complementary DNA	Resonance light scattering (RLS)	50 pM to 300 nM	6.1 pM	[188]
miR122	Electroactive Prussian Blue (PB) nanoparticles grown on graphene oxide (GO), which is anchored by Au-linked target complementary DNA	miRNA Complementary DNA	DPV	10 fM to 10 nM	1.5 fM	[86]
miR-122, miR-223, and miR-21	DNA-conjugated gold nanoparticles, F-AuNPs (probe), and DNA-conjugated Ag-coated magnetic nanoparticles, AgMNPs (capture)	Complementary DNA	SERS	1 fM to 100 nM	349 aM (miRNA-122), 374 aM (miRNA-223) 311 aM (miRNA-21)	[87]
miR-16	Dual-aptamer hairpin DNA oligonucleotide to bind to miRNA	Complementary DNA	DPV	50 and 2000 nM	0.14 nM	[88]
miR-21 and miR-141	Multifunctional Fe_3_O_4_magnetic nanoparticles modified cDNA probe	Complementary DNA	DPV	1 fM to 1 nM	0.36 fM (miR-21) 0.28 fM (miR141)	[89]
miR-21	Target-linked silicon magnetic beads	Complementary DNA	SERS	-	0.3 fM	[90]
miR-34a	Zip nucleic acid-immobilized streptavidin-coated magnetic beads (MBs)/pencil graphite electrode to capture the target miRNA/miRNA-DNA duplex	Zip nucleic acid	DPV	10 to 30 µg/mL	0.87 µg/mL	[91]
miR-133a	U-shaped biosensors utilizing wavelength shift and transmission loss	Complementary DNA	SPR combined with immunoassay	0.286 to 0.0133 ng/mL	0.0133 ng/mL	[92]
miR-222	AuNps self-assembled cDNA probe of target miRNA on rGO-modified glassy carbon electrode (GCE)	Complementary DNA	DPV	(0.5 fM to 70 nM	0.03 fM	[95]
HULC	Au@Ag core–shell nanoparticles/graphene quantum dots (Au@Ag/GQDs)	Complementary DNA	ECL	1 fM to 5 nM	0.3 fM	[96]
miRNA	Fe_3_O_4_ NPs, indium tin oxide cathode, and a graphene oxide/gold nanoparticle/glucose oxidase anode	Complementary DNA	Biofuel cell method	10 aM to 10 fM	1.4 aM	[97]
miR-141	(dsDNA)-templated copper nanoparticles (CuNPs) immobilized on gold surface electrode-T7 exonuclease (exo)-assisted cascade digestion method	Complementary DNA-probes	DPV	10^−11^ to 10^−16^ M	4.5 × 10 ^−17^ M	[98]
miR-21	Circle capture probe anchored at the top of the tetrahedron DNA nanostructure (TDN)	Complementary DNA-probes	SWV	0.1 fM to 10 nM	18.9 aM	[99]
miR-155	Electrochemical genosensor for the detection of miRNA-21 and miRNA-155 using a tetrahedron DNA nanostructure (TDN	Complementary DNA-probes	SWV	0.1 fM to 10 nM	39.6 aM	[99]
miR-122	Hybridization chain reaction (HCR) and hairpin DNA (hpDNA)	Complementary DNA-probes	DPV	0.1 fM to 0.1 μM	53 aM	[100]
	Strand displacement amplification (SDA) reaction and analogical catalytic hairpin assembly (ACHA) reaction	Complementary DNA-probes	DPV	0.1 fM to 10 fM	0.012 fM	[101]
miR-21	Strand displacement amplification (SDA) reaction and analogical catalytic hairpin assembly (ACHA) reaction	Complementary DNA-probes	DPV	0.1 fM to 10 fM	0.075 fM	[101]
	Hybridization chain reaction (HCR) amplification strategy	Complementary DNA-probe	EIS	10 fM to 50 pM	4.63 fM	[102]
	Ratiometric electrochemical DNA biosensor is based on a locked nucleic acid (LNA)-modified “Y” shape-like structure	DNA-probe	DPV	10 fM to 70 fM	2.3 fM	[103]
	DNA nanospheres, nicking endonuclease-assisted primer exchange reaction (PER) cascade amplification	DNA-probe	DPV	1 aM to 0.1 nM	0.58 aM	[104]
	QCM sensor surface immobilized with pyrene DNA intercalators	Gold-nanoparticle-conjugated DNA	QCM	2.5 pM to 2.5 μM	3.6 pM	[105]
	Gold nanoparticles (AuNps), a surface-immobilized capture oligonucleotide	DNA-probe	QCM and SPRi microarray	(0.1–50 pM)	QCM: 28 fM SPRi: 47 fM	[106]
Methylated DNA	Restriction endonuclease (DPnI)/hairpin DNA (HP)	CRISPR/Cas12a *trans*-cleavage activity	ECL	5–70 U/mL	23.4 mU/mL	[70]
	Stem-loop–tetrahedron composite DNA/Au-nanoparticle-coated gold electrode	Stem-loop–tetrahedron composite DNA probe	Chronoamperometry	1 aM to 1 pM	0.075 pM	[71]
**Protein Biomarkers**						
Alpha-L-Fucosidase (AFU)	Target activity on 2-chloro-4-nitrophenol (2-chloro-4-NP)	PVC membrane sensor	Potentiometry	10^−2^–10^−5^ M	1.0 × 10^−6^ M	[115]
	Carbon dots (C-dots) and gold nanoparticles (AuNPs)	Antibody	FRET	11.3 to 200 nM	3.4 nM	[117]
Glypican-3 (GPC-3)	Glassy carbon electrode-modified NP-conjugated antibody magnetite (Fe_3_O_4_) nanoparticles (NPs) decorated with hyperbranched amino-functionalized dendrimers	Antibody	CV and EIS	0.02 to 10 ng/mL	70 pg/mL	[121]
	GPC3-aptamer-labeled gold carbon dots (AuCDs-GPC3Apt) and magnetic graphene oxide (Fe_3_O_4_/GO) nanosheets	Aptamer	FRET	5–100 ng/mL	3.01 ng/mL,	[122]
	Combining hemin-reduced graphene oxide-platinum nanoparticles with reduced graphene oxide–gold nanoparticles	Aptamer	EIS	0.001 μg/mL to 10 μg/mL	0.001 μg/mL	[123]
	Platinum@palladium nanoparticles decorated with hemin-reduced graphene oxide (H-rGO-Pt@Pd NPs)	Aptamer	DPV	0.001 μg/mL to 10 μg/mL	0.181 ng/mL	[124]
	Reduced graphene oxide-hemin nanocomposites (RGO-hemin) modified on the screen-printed electrode surface	Aptamer	DPV	0.00 1–10.0 µg/mL	2.86 ng/mL	[125]
	Hemin/graphene nanohybrid (HGN)-aptamer: catalytic silver deposition	Aptamer	DPV	10.0–100.0 μg/mL	3.16 μg/mL	[126]
Alpha-fetoprotein (AFP)	AFP-aptamer-labeled luminescent CdTe quantum dots (QDs) and anti-AFP antibody functional gold nanoparticles (AuNPs)	Aptamer	FRET	0.5–45 ng/mL	400 pg/mL	[94]
	Gold nanoparticles-dextran-reduced graphene oxide (AuNPs-Dex-RGO)-AuNps	Antibody	DPV	0.01–20 ng/mL	0.05 pg/mL	[95]
	Polyethylene glycols (PEG)/gold nanoparticles (AuNPs)/polyaniline (PANI): PEG/AuNPs/PANI composite	Antibody	DPV	0.01 pg/mL –1.0 ng/mL	0.007 pg/mL	[133]
	AgNPs-Ab probes	Antibody	(NIE)	N/A	5 pg/mL	[189]
	Au/CsxWO3 heterogeneous films	Antibody	photoelectrochemical biosensor	0.01 ng/mL to 500 ng/mL	7 pg/mL	[138]
Golgi protein 73 (GP73)	CdSe quantum dots, modified by aleuria aurantia lectin (AAL) and PEG, as a substrate. A glassy carbon electrode, modified with electrochemically reduced graphene oxide	Antibody	EIS	m 20 to 5000 pM	12 pM	[140]
	Nitrogen-doped graphene quantum dots (NGQDs) labeled with GP73 aptamer (GP73Apt) and molybdenum disulfide @ reduced graphene oxide (MoS2@RGO) nanosheets	Aptamer	FRET	5 ng/mL–100 ng/mL	4.54 ng/mL	[144]
	Manganese-modified CdTe/CdS quantum dots	Antibody	Fluorescence spectroscopy	20–150 ng/mL	10 ng/mL	[145]
	Reduced graphene oxide-carboxymethyl chitosan-hemin/platinum@palladium nanoparticles (RGO-CMCS-hemin/Pt@Pd NPs)	Aptamer	Colorimetric aptasensor	10.0–110.0 ng/mL	4.7 ng/mL	[146]
	DNA tetrahedron nanostructure (DTN)-modified electrode and (Ru(dcbpy)_3_Cl_2_) used for ECL	Antibody	ECL	15 pg/mL–0.7 ng/mL	15 pg/mL	[147]
	nitrogen-doped graphene quantum dots (N-GQD_S_) and molybdenum disulfide (MoS_2_) nanosheets	Aptamer	FRET	2.5 ng/mL∼100 ng/mL	4.54 ng/mL	[148]
	Hemin-reduced graphene oxide–manganese oxide (H-rGO-Mn_3_O_4_) nanozymes/Gold@poly(o-phenylenediamine) (Au@POPD) nanohybrids	Aptamer	CV	0.01–100.0 ng/mL	0.0071 ng/mL	[148]
	Streptavidin magnesphere paramagnetic particles (PMPs)-methylene blue (MB)-labeled DNA probe/nafion-modified indium tin oxide electrode	Antibodies	PLA and enzyme-powered recycling amplification Electrochemical immunosensor	0.3 pg/mLto 6.0 ng/mL	0.10 pg/mL	[149]
	streptavidin-labeled MNPs/biotin-labeled monoclonal antibody, (AE)-labeled reporter monoclonal antibody	Antibodies	Chemiluminescence	1.34 ng/mL to 684.38 ng/mL.	1.19 ng/mL	[150]
Osteopontin (OPN)	Lateral-flow-pad-immobilized OPN antibody/aptamer complementary/AuNPs-SA conjugates	Antibody and aptamer	Lateral flow aptasensor	10–500 ng/mL	0.1 ng/mL	[147]
	RNA-aptamer-modified gold electrode surface of a strip containing a silver pseudo-reference electrode and a gold counter-electrode	Aptamer	CV	25 nM and 2402 nM	520 ng/mL	[155]
	DNA-aptamer-modified electrode	Aptamer	CV/SWV	12 to 1540 nM	1.3 ± 0.1 nM.	[156]
	ZrO2@GNF nanohybrids with different morphologies/nanostructures derived from zirconium-based metal–organic frameworks (UiO-66) entrapped within the electric spun polyacrylonitrile (PAN)	Aptamer	EIS	0.01 pg/mL to 2.0 ng/mL	4.76 ng/mL	[160]
	Nanohybrid composed of Ti_3_C_2_T_x_ MXene and phosphomolybdic acid (PMo12) embedded with polypyrrole (referred to as PPy@ Ti_3_C_2_T_x_/PMo12)	Aptamer	EIS	0.05–10,000 pg/mL	0.98 fg/mL	[159]
Squamous cell carcinoma antigen (SCCA)	β-cyclodextrin-functionalized graphene nanosheet (CD-GN)/ternary hollow Pt/PdCu nanocube anchored on three-dimensional graphene framework (Pt/PdCu-3DGF)	Antibody	CV	0.0001 to 30 ng/mL	25 fg/mL.	[162]
	Au-NPs@Zn-MOF functionalized with 1H-imidazolium1,3-bis(2-aminoethyl)bromide ionic liquid (IBABr), IBABr-Au@Zn-MOF nanocomposites	Antibody	PEC	5.0 pg/mL to 15.0 ng/mL	2.34 pg/mL	[163]
	Triangle-shaped silver nanoparticle array was fabricated using nanosphere lithography modified with MUA and antibody	Antibody	LSPR	0.1–1000 pM	0.125 pM	[164]
	Carboxyl-functionalized CdS nanoparticles (CdS NPs) bonded onto Fe-TiO_2_ modified with antibody	Antibody	PEC	0.001 ng/mL to 75 ng/mL	0.22 pg/mL	[165]
	BiOBr/Bi2S3/ascorbic acid (AA)/antibody-modified ITO electrode	Antibody	PEC	0.001–75 ng/mL	0.3 pg/mL	[166]
Hedgehog (Hh) ligands	Texas-Red-labeled AP32 to microbead	Aptamer	Fluorescence spectroscopy	0.07 to 62.5 nM	69 pM	[168]
	Aptamer specific to SHh and the combination of primer exchange reaction (PER) and catalytic hairpin assembly (CHA)	Aptamer	CV	N/A	4.1 pM	[169]
Beta-catenin	Peptide-based probes		SWV	32 pM to 10 nM	<10 pM	[172]
Aflatoxin B1 (AFB1)	MWCNTs/RTIL composite films	Antibody	EIS	0.1–10 ng/mL	0.03 ng/mL	[157]
Lipocalin-2 (LCN2)	Single-stranded DNA aptamer pairs	Aptamer	SPR	2.5–500 ng/mL	0.6 ng/mL	[176]
Pentraxin-3 (PTX-3)	Functionalized multi-walled carbon nanotubes (MWCNTs) and gold nanowire (AuNW) nanocomposites	Antibody	DPV	0.001–1000 ng/mL	0.16 pg/mL	[178]
Amyloid-beta protein	Gold nanoparticles-functionalized graphitic carbon nitride nanosheets (g-C3N4@Au NPs) and palladium nanoparticles-coated metal–organic framework (Pd NPs@NH2-MIL-53)-modified glassy carbon electrode (GCE)	Antibody	ECL	0.01 pg/mL to 50 ng/mL	3.4 fg/mL	[181]

## 6. Conclusions and Future Perspectives

The concept of the early-stage detection of tumors is to prevent their spread to other organs and becoming incurable. HCC is one of the significant threats to human health and a heavy economic burden for nations. In this review, we have highlighted the recent biosensor developments for the early-stage detection of HCC. The detection methods are based on detecting HCC-specific biomarkers, such as proteins, nucleic acids, metabolites, small molecules, exosomes, and cancer cells, to monitor the progression of HCC for high-risk groups. Using AFP as a primary protein biomarker for HCC detection and surveillance is common practice. Nonetheless, this marker is suboptimal for monitoring the responses to the treatment. Therefore, due to the high mortality and poor prognosis of HCC, it is considered an inadequate candidate for the early-stage diagnosis of HCC. Several biomarkers have the potential to diagnose HCC in its early stages. Liquid biopsy is used to detect and analyze CTCs, ctDNA, exosomes, miRNA, and lncRNA, which implies a better understanding of genetic modification and mutations for the diagnosis of early-stage liver tumors. Another advancement in biomarker detection is the use of advanced technology, such as next-generation sequencing, which would undoubtedly significantly improve the discovery of new biomarkers specific to HCC. A recent review by Erkocyigit et al. highlighted the application of paper-based microfluidic analytical devices (μPADs) for the point-of-care applications. They used serum samples for the diagnosis of cancer biomarkers using different types of biosensors [190]. Another report by Kim et al. analyzed 609 patient samples. The samples were tested for ctDNA (mutated TP53, methylated RASSF1a, and GSTP1) and AFP from urine using a two-stage model with 79% sensitivity and 90% specificity, which is 30% more than the case detected from AFP alone [191]. Multiple biomarker-specific ssDNA-aptamer-conjugated superparamagnetic iron oxide nanoparticles (SPIONs) are used as signal enhancers for the accurate diagnosis of cancer by Magnetic Resonance Imaging (MRI) [19]. Despite many biomarker discoveries for HCC diagnosis, implementing these candidates for clinical diagnosis is challenging. The reported studies used only a limited number of real samples for validation, which is insufficient for reliability and accuracy. Moreover, a substantial number of optimization steps are carried out for each biomarker to be used for diagnostic and prognostic purposes. Additionally, advanced nanomaterials need to be incorporated into the existing methodology to improve the sensitivity and specificity of the sensing device. Furthermore, the new methodology must recognize the target biomarkers with the minimum quantity to validate the sample for the diagnosis and prognosis of HCC.

## Abbreviations

HCC	Hepatocellular carcinoma
AFP	Alpha-fetoprotein
CTSs	Circulating tumor cells
MiRNA	microRNAs
PET	Positron Emission Tomography
CT	Computed Tomography
MRI	Magnetic Resonance Imaging
MRS	Magnetic Resonance Spectroscopy
ctDNA	Circulating tumor DNA
ddPCR	Digital droplet polymerase chain reaction
TP53	Tumor Protein 53
CTNNB1	Catenin beta-1
BiFC	Bimolecular fluorescence complementation
TCF3	Transcription factor 3
TCF3ΔNLS	TCF3 lacking the nuclear localization signal
TERT	Telomerase Reverse Transcriptase
HBV	Hepatitis B virus
HCV	Hepatitis C virus
NGS	Next-generation sequencing
WGS	Whole-genome sequencing
Cap-Seq	Capture sequencing (Cap-seq)
cfDNA	Cell-free deoxyribonucleic acid
ccfDNA	Circulating cell-free DNA
WTB-PCR	Wild-type blocking polymerase chain reaction
CHIP	Clonal hyperplasia with indeterminate potential
ATM	Ataxia telangiectasia mutated
FRET	Fluorescence resonance energy transfer
CpGs	Carbon-5 cytosine–guanine dinucleotide
MS-HRM	Methylation-sensitive high-resolution analysis
LDHB	Lactate Dehydrogenase B
qRT-PCR	Quantitative real-time polymerase chain reaction
AuNps	Gold nanoparticles
ECL	Electrochemiluminescence
Dam MTase	DNA adenine methylation methyltransferase
PNA	Peptide nucleic acid
PB	Prussian Blue
GO	Graphene oxide
RGO	Reduced graphene oxide
SERS	Surface-enhanced Raman scattering
CHA	Chain hybridization amplification
HCR	Hybridization chain reaction
CuNPs	Copper nanoparticles
MB	Methylene blue
DPV	Differential pulse voltammetry
CV	Cyclic voltammetry
SWV	Square wave voltammetry
EIS	Electrochemical impedance spectroscopy
TDN	Tetrahedron DNA nanostructure
SDA	Strand displacement amplification
ACHA	Analogical catalytic hairpin assembly
LNA	Locked nucleic acid
DNA	DNA nanosphere
QCM	Quartz crystal microbalance
LOD	Limit of detection
GPC-3	Glypican-3
GMR	Giant magnetoresistive effect
GP73	Golgi protein 73
PEG	Polyethylene glycol
AAL	Aleuria aurantia lectin
TMB	3,3′,5,5′-Tetramethylbendzidine
DTN	Tetrahedron nanostructure
SA	Streptavidin
NGQDs	Nitrogen-doped graphene quantum dots
PLA	Proximity ligation assay
GPC-3	Glypican-3
OPN	Osteopontin
SELEX	Systematic evolution of ligands by exponential enrichment
PAN	Polyacrylonitrile
SWCNTs	Single-walled carbon nanotubes
MWCNTs	Multi-walled carbon nanotubes
PMol2	Phosphomolybdic acid
SCCA	Squamous cell carcinoma antigen
SPR	Surface Plasmon Resonance
LSPR	Localized Surface Plasmon Resonance
SPRi	Surface Plasmon Resonance Imaging
PEC	Photoelectrochemical
Hg	Hedgehog
SHh	Sonic hedgehog
PER	Primer exchange reaction
CHA	Catalytic hairpin assembly
KLK6	Kallikrein 6
LCN2	Lipocalin-2
PTX-3	Pentraxin-3
ATRP	Atom transfer radical polymerization
GCE	Glassy carbon electrode
QCM-D	Quartz crystal microbalance with dissipation monitoring
2-AP	2-Aminopurine
RLS	Resonance light scattering
NIE	Nanoimpact electrochemistry
